# Perfluorocarbon emulsion enhances MR-ARFI displacement and temperature *in vitro*: Evaluating the response with MRI, NMR, and hydrophone

**DOI:** 10.3389/fonc.2022.1025481

**Published:** 2023-01-13

**Authors:** Ryan Holman, Orane Lorton, Pauline C. Guillemin, Stéphane Desgranges, Francesco Santini, Davide Bernardo Preso, Mohamed Farhat, Christiane Contino-Pépin, Rares Salomir

**Affiliations:** ^1^ Image Guided Interventions Laboratory (GR-949), Faculty of Medicine, University of Geneva, Geneva, Switzerland; ^2^ Avignon Université, Equipe Systèmes Amphiphiles bioactifs et Formulations Eco-compatibles, Unité Propre de Recherche et d'Innovation (UPRI), Avignon, France; ^3^ Department of Biomedical Engineering, University of Basel, Basel, Switzerland; ^4^ Institute of Mechanical Engineering, Ecole Polytechnique Fédérale de Lausanne, Lausanne, Switzerland; ^5^ Radiology Department, University Hospitals of Geneva, Geneva, Switzerland

**Keywords:** HIFU, hydrophone, emulsion, sonosensitizers, MR-ARFI, colloids, perfluorocarbons (PFCs), cavitation

## Abstract

Sonosensitive perfluorocarbon F_8_TAC_18_-PFOB emulsion is under development to enhance heating, increase thermal contrast, and reduce treatment times during focused ultrasound tumor ablation of highly perfused tissue. The emulsion previously showed enhanced heating during *ex vivo* and *in vitro* studies. Experiments were designed to observe the response in additional scenarios by varying focused ultrasound conditions, emulsion concentrations, and surfactants. Most notably, changes in acoustic absorption were assessed with MR-ARFI. Phantoms were developed to have thermal, elastic, and relaxometry properties similar to those of *ex vivo* pig tissue. The phantoms were embedded with varying amounts of F_8_TAC_18_-PFOB emulsion or lecithin-PFOB emulsion, between about 0.0-0.3% v:w, in 0.05% v:w increments. MR-ARFI measurements were performed using a FLASH-ARFI-MRT sequence to obtain simultaneous displacement and temperature measurements. A Fabry-Perot hydrophone was utilized to observe the acoustic emissions. Susceptibility-weighted imaging and relaxometry mapping were performed to observe concentration-dependent effects. ^19^F diffusion-ordered spectroscopy NMR was used to measure the diffusion coefficient of perfluorocarbon droplets in a water emulsion. Increased displacement and temperature were observed with higher emulsion concentration. In semi-rigid MR-ARFI phantoms, a linear response was observed with low-duty cycle MR-ARFI sonications and a mono-exponential saturating response was observed with sustained sonications. The emulsifiers did not have a significant effect on acoustic absorption in semi-rigid gels. Stable cavitation might also contribute to enhanced heating.

## Introduction

Magnetic resonance-guided focused ultrasound (MRgHIFU) is a noninvasive ablative procedure that allows reduced morbidity and faster recovery for certain medical treatments. Under MRI guidance, the operator can monitor the temperature change in each voxel to ensure that the cancer cells were sufficiently heated to induce apoptosis. This allows accurate determination of positioning, in addition to providing more certainty to a sufficient thermal dose. The temperature increase generated by the focused ultrasound beam reduces the amount of hydrogen bonding in the water molecules, reducing the resonance frequency of these atoms, resulting in a temperature-dependent shift in proton spectra (1–3), that generates a measurable change in the phase maps of MRI images (4, 5). Commercial magnetic resonance thermometry (MRT) sequences allow monitoring of intraoperative temperature changes using this principle.

Alternative experimental MRI sequences are of interest to monitor HIFU procedures for other applications. Magnetic resonance acoustic radiation force impulse (MR-ARFI) imaging can measure the simultaneous increase in temperature and displacement from a focused ultrasound (FUS) pulse (6–8), with displacement increasing linearly with applied ultrasound power (8, 9). The technique is used for applications, including distinguishing healthy tissue or diseased tissue, quantifying tissue stiffness, identifying ablation lesions, focal point tracking without heat damage, adjusting for phase aberrations, and focal spot shaping (10–15). MR-ARFI has been implemented in pilot studies, such as for noninvasive breast palpation before and after focused ultrasound lesion ablation (10) and for transcranial displacement measurements (12). Simulation of the MR-ARFI displacement can be performed with finite-difference solutions to equations of motion (16–18) or by convolution techniques (15, 19). Not least of all, the MR-ARFI sequence has potential for focal spot tracking during localized, targeted, low-temperature focused ultrasound-mediated sonoporation drug delivery. Perfluorooctyl bromide (PFOB) nanoemulsions have shown effective *in vitro* and *in vivo* for non-thermal and non-cavitational drug delivery (20, 21). Clinical studies for focused ultrasound-mediated drug delivery in liver tumors with liposomal doxorubicin formulations (ThermoDox, Celsion Corp, Lawrenceville, New Jersey, USA) reported improved tumoral doxorubicin concentrations (22, 23).

Focused ultrasound is indicated for ablation in a variety of tumor types. However, the procedure faces certain limitations, particularly for hepatobiliary treatments (24). Notable impediments in liver ablation include interactions with the thoracic cage and respiratory motion (25–28). Additional measures are often required, such as anesthesia, intubation, and rib resection (29, 30). The left liver lobe is exposed for a portion of the respiratory cycle allowing ablation to be performed, although the right liver lobe is predominantly located behind the rib cage which complicates the treatment (31). Also, high-risk tumor sites are located near to adjacent organs, particularly near the gallbladder and bowels. Retrospective review has given common side-effects in hepatobiliary ultrasound-guided FUS procedures to be skin burns, pain, and fever at 15%, 5%, and 2%, respectively (29). Though, more adverse events have been reported, including biliary obstruction, fistula formation, osteonecrosis, and diaphragmatic rupture (30). A variety of techniques were developed to overcome some limitations of the procedure including rib shielding devices (32), a large acoustic window super-focusing transducer dedicated to intra-abdominal treatments (33), and techniques to account for respiratory motion (34–37).

The use of exogenous cavitation nuclei is particularly interesting for focused ultrasound-mediated blood-brain barrier (BBB) opening to enhance drug delivery in brain tumors. Low-intensity MRgFUS in combination with microbubbles can increase BBB permeability, by temporarily and reversibly disrupting these pathways, allowing tumoral accumulation of higher amounts of standard chemotherapeutics and larger molecular weight chemotherapeutics that would otherwise be incapable of paracellular transport (38). Microbubbles typically exhibit a lower threshold for cavitation than droplets, as the droplets must first undergo vaporization, whether in the perfluorocarbon or in the surrounding medium, to promote further gas accumulation (39). Microbubble contrast agents also have a particular advantage in human studies in that these can be used off-label in neuro-oncological therapeutic treatments. This emulsion formula, at reduced droplet size, has shown capable as an *in vivo* drug delivery vehicle in combination with microbubbles for focused ultrasound-mediated BBB disruption (21).

In previous studies, perfluorocarbon nanoemulsions were developed for theranostic applications, both as a chemotherapy drug carrier and ^19^F-MRI contrast agent (40). The formula was later adapted for ablative HIFU applications. Micron-sized F_8_TAC_18_-PFOB droplets were designed for intra-capillary transpulmonary circulation, illustrating repeatability and enhanced heating in a tissue mimicking material (TMM) phantoms with focused ultrasound (41). Subsequent experiments in *ex vivo* pig kidneys and liver showed an increased energy deposition by a factor of 3, for a 0.24% v:v solution of 1.42-2.16 ± 0.68 µm in the perfused fluid, at flow rates up to 0.15 mL.s^-1^, capable of repeatable sonication without rupture or embolism (42). The acoustic absorption coefficient of a TMM phantom perfused with a solution of this perfluorocarbon emulsion was observed to scale mono-exponentially with concentration to an asymptotic maximum, at doses expected safe for human use (43). The results gave insight into the size of the probability of temperature increase around an individual droplet, which was denoted as the interaction radius. This observation allowed development of a simulated model to predict an interaction radius of 12.5 µm for droplets with diameters of 1.9-2.3 µm.

In this study, experiments evaluated whether TMM gel phantoms exhibited an increase in the displacement of MR-ARFI due to perfluorocarbon droplets and whether cavitation was present. It was hypothesized that enhanced absorption could also increase displacement. Further measurements were performed with MRI, NMR, hydrophone, and rheometry to characterize the droplets and TMM phantoms. To the authors’ knowledge, this is the first study using MR-ARFI to evaluate acoustic absorption changes from sonosensitive droplets. Additionally, a TMM gel phantom formula was further developed that was capable of use with microscale MR-ARFI displacement measurements and MR thermometry with higher-temperature sustained sonications. Moreover, new details were gained on the acoustic absorption mechanism of this particular perfluorocarbon emulsion formula.

## Methods

### Emulsion preparation

Materials included PFOB (Fluorochem, Hadfield, United Kingdom), lecithin (90%, soy bean, 76870, Thermo Fisher, Kandel, Germany), 1H,1H,2H,2H-perfluorooctanethiol (Atochem, Colombes, France), with all other reagents (sodium trifluoroacetate, AIBN) and solvents being reagent grade. Two surfactants were employed to stabilize the emulsion, lecithin and an in-house fluorinated surfactant named F_8_TAC_18_, giving rise to the lecithin-PFOB or F_8_TAC_18_-PFOB emulsions, respectively.

The F_8_TAC_18_ surfactant consists of a water-soluble oligomer of Tris(hydroxymethyl)aminomethane (Tris), which constitutes the polar head of the surfactant, and a perfluorinated tail group (44, 45), that allows tuning the droplet size by altering the number of dimethylene groups in the perfluorinated tail group. The F_8_TAC_18_ surfactant and emulsions were prepared as described in previous publications (40–43, 46). In brief, a low energy process was employed to prepare the emulsions using a homogenizer system (Polytrons PT 3100, Kinematica, Luzern, Switzerland). The general routine procedure consisted of dissolving 500 mg of emulsifier in 196 mL of water, after which 4 mL of PFOB was added. The solution was then cooled in an ice bath. The solution was homogenized three times for 15 minutes at 20,000 rpm for the lecithin emulsion. Similarly, the solution was homogenized three times for 15 minutes at 24,000 rpm for the F_8_TAC_18_ emulsion. The emulsions were then refrigerated at 4 °C until use.

The particle size distributions were determined by laser diffraction and Mie light scattering theory, using a Mastersizer 2000 with a Hydro2000S dispersion unit module (Malvern Instruments, Orsay, France). The dispersant solution consisted of water with a refractive index of 1.333, while the refractive index of PFOB was 1.305. The emulsions were added to the Hydro2000S dispersion unit while stirring at 18,550 rpm. Then the system formulated the volume-weighted mean diameter (D[4,3]). The volume fraction of PFOB in the solution was determined with ^19^F nuclear magnetic resonance (NMR), as described in previous studies (46). The D[4,3] was 1.83 µm for the lecithin droplets studies. The D[4,3] ranged between 1.70-1.76 µm for the F_8_TAC_18_ droplets, except one sample which was 1.24 µm.

### Fabrication of TMM phantoms

**Figure 1:**
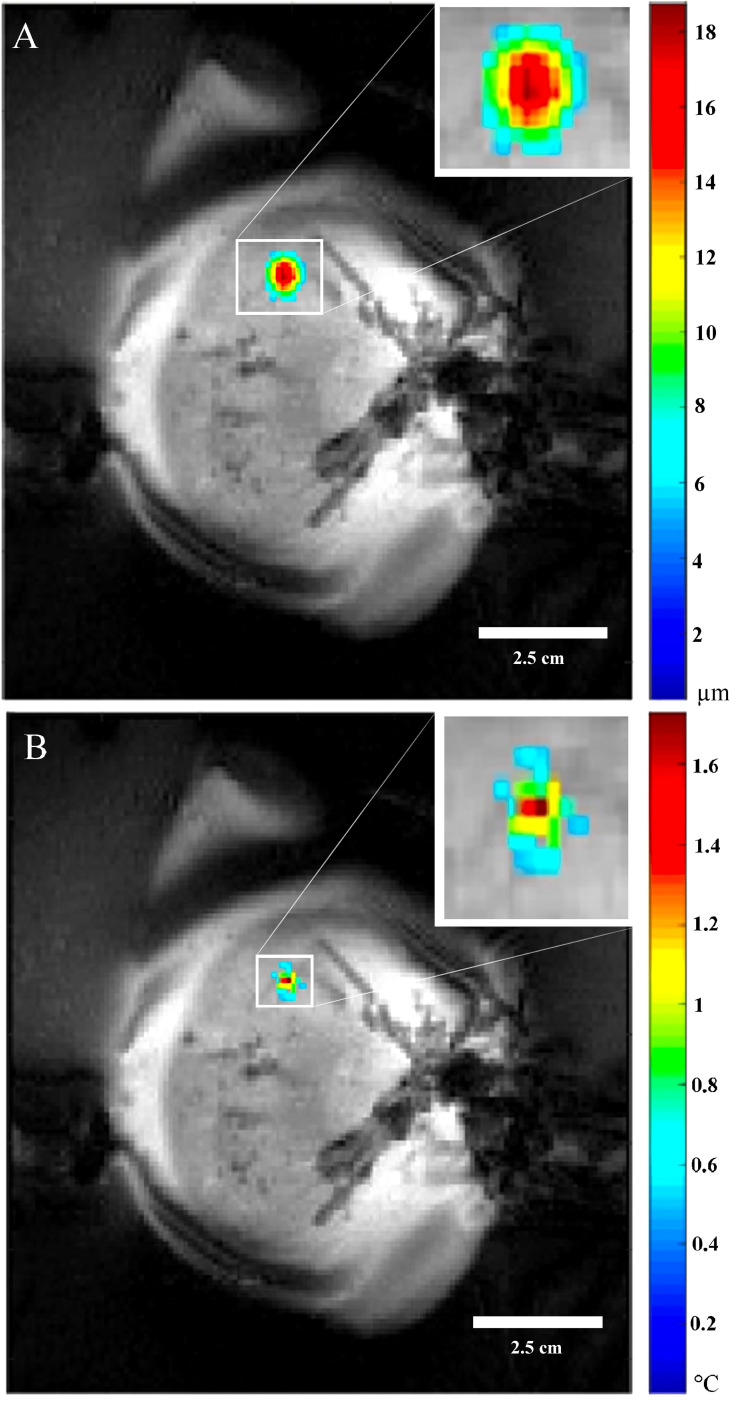
MR-ARFI focal spot in the insonicated area. **(A)** MR-ARFI *ex vivo* porcine kidney displacement overlaid on the magnitude map. **(B)** MR-ARFI *ex vivo* porcine kidney temperature elevation overlaid on the magnitude map. The insets are higher magnification to provide more detail of the focal spot.

The phantom formulas were made using modifications to previously described MR-ARFI phantoms (47, 48). The TMM phantom formulas were adjusted to emulate *ex vivo* pig tissue properties, by comparing temperature and displacement measurements in *ex vivo* pig kidney, pig liver, and pig myocardium. An example of MR-ARFI temperature and displacement measurements in an *ex vivo* pig kidney is shown in **Figure 1**. The figure shows the displacement and temperature maps generated during MR-ARFI sonications overlaid on the MRI magnitude images in the location of the insonicated area. These values were used for comparison with subsequent iterations of TMM sample formulations, using equivalent sonication conditions. The figure also provided a good depiction of how the focal spot displacement and temperature profiles appear relative to mammalian anatomical features.

The proportions of starch, condensed milk, gelatin, and agarose were adjusted until the phantom provided similar MR-ARFI displacement and temperature response as seen in *ex vivo* pig organs. Agarose was later determined to make the phantoms too inelastic, and the substance was omitted from the formula. Some formulas, such as those incorporating agarose, tended to give good thermal response, but the elastic properties were too stiff, and displacement could not readily be measured. Alternatively, with other formulas, good elastic properties were obtained, but the phantoms would then melt under higher power sustained sonications. Two emulsifiers were tested: lecithin and F_8_TAC_18_. The final gel formula was 143 grams total, composed of 3% w:w gelatin, 6% w:w starch (maize flour), 5% w:w powdered milk (containing 36% w:w proteins, 0.8% w:w fats), and 0.021% w:w sodium azide as a preservative, between 0.05-0.3% v:w PFOB emulsion, and the remainder of approximately 86% v:v degassed and deionized water. Powdered milk at 10% w:w was used in certain gel samples, for powdered milk containing 18% w:w proteins. Also, benzalkonium chloride (BAL) was used as a preservative in lieu of sodium azide on occasion. Two control gels were made simultaneously, free of any PFOB emulsion. ^19^F-NMR was used to verify the concentrations of selected gel sets. Phantoms used both emulsion formulas near 0.05% v:w, 0.1% v:w, 0.2% v:w, and 0.3% v:w. An example of quantitative ^19^F-NMR measurements for PFOB concentration is available in the supplementary materials.

**Figure 2:**
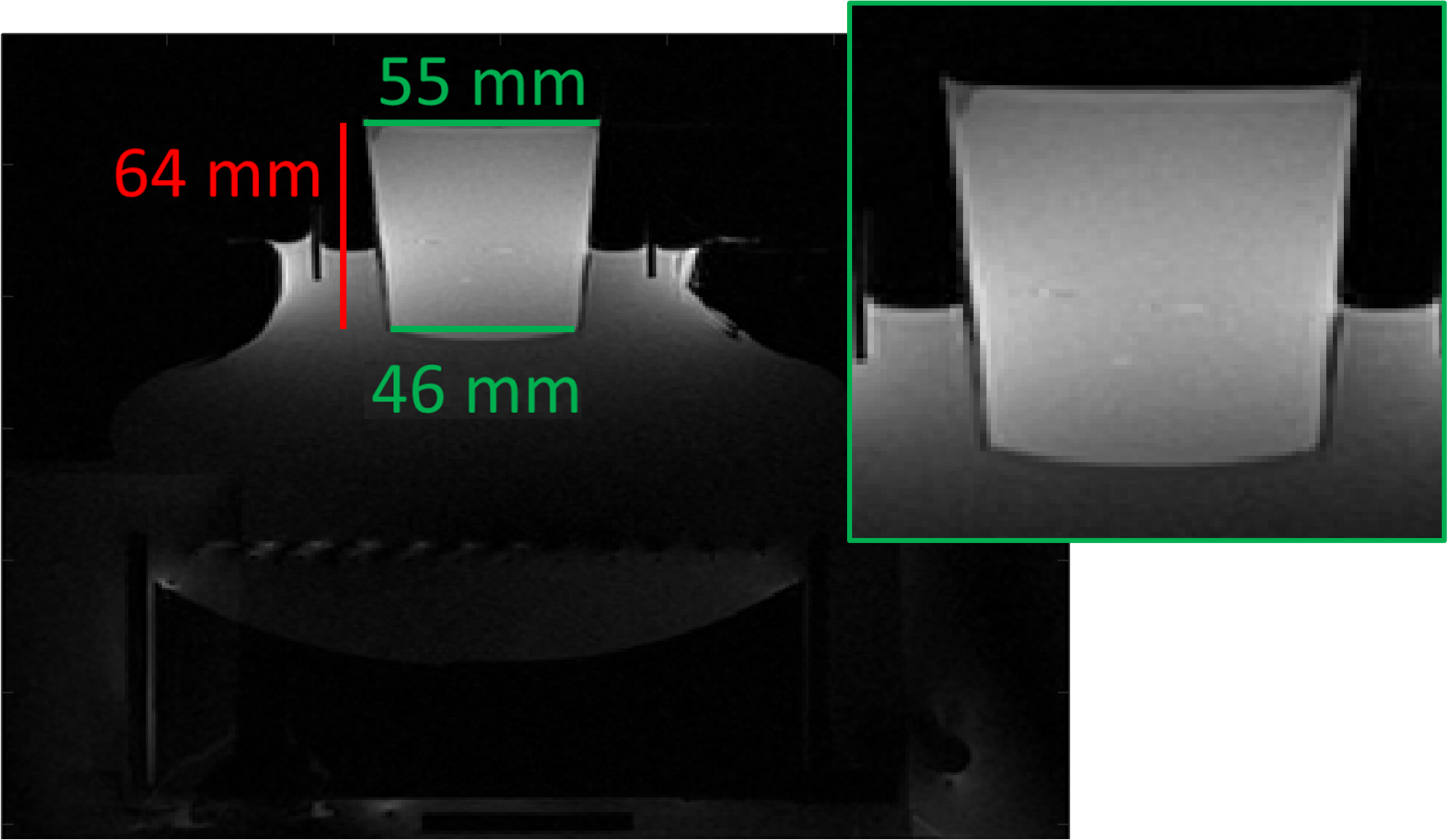
Illustration of TMM gel phantom placed on top of the MRgHIFU transducer system. This MR image is a transverse view generated from a T1-VIBE sequence. The inset shows an enlarged view of the TMM phantom. Also visible in the image is the transducer and coupling components.

A protocol to fabricate the TMM gel sets with this final formulation can be found in the supplemental materials. Briefly, degassed and deionized water was heated to boiling, then the heat was removed. Then added to the hot water was condensed milk, starch, and gelatin. The solution was mixed homogeneously and cooled at room temperature to about 65 °C before being mixed with the emulsion solution. The phantoms were cast into two plastic cups, one placed within the other. The bottom of each plastic cup was removed, and a thin piece of food cellophane was placed between the cups, to create a smooth, watertight surface. The warm liquid gel was poured into this container, allowed to cool at room temperature for about one hour, placed in an ice bath to solidify, then stored in the refrigerator at 4 °C. An MR image of a TMM gel sample is shown in **Figure 2**. The size varied slightly on occasion, as some samples were fabricated at different facilities, with different inventory of equipment. However, the important aspect was for the size of the TMM gel phantom to be large enough, relative to the size of the focal spot, to negate effects of ultrasound reflections at the TMM surfaces from influencing the measurements. Ultrasound coupling gel was further placed on top of the TMM phantom to prevent ultrasound waves reflecting at the surface but was not applied until after this image was acquired, just prior to performing the sonications.

Gas entrapment was found to occur when the emulsion was mixed too vigorously and when the gel was cooled too quickly. Measures were taken to reduce gas entrapment. First, degassed and deionized water was used in the fabrication of the gel sets. The degassing was done by circulating deionized water through a dedicated degassing unit, using a vacuum pump and a semi-permeable membrane-based gas extraction. The dissolved oxygen content was then measured with a fluorescence probe (Neofox, Ocean Insight, Orlando, FL, USA). The emulsion was also dispersed with a syringe and a needle, with the tip submerged in the liquid gel solution. The syringe was further used to mix the TMM solution with repeated cycles of drawing and dispensing, rather than with manual stirring. Additionally, the gels were cooled at room temperature for a prolonged time, prior to solidifying the gel sets in an ice bath, to allow entrapped air bubbles to naturally collect at the top surface. These methods help reduce the complications of unintentional atmospheric gases being entrapped in the TMM gel matrix.

### Focused ultrasound system

The incident high intensity focused ultrasound (HIFU) sonications were generated by an acoustic field that was pulsed according to the duty cycle settings, at an acoustic frequency of 1.031 MHz. The transducer was a pseudorandom array consisting of 256 elements, 131 mm focal length, and 140 mm aperture. The transducer was connected to a beam former and impedance matching unit (Image Guided Therapy, Pessac, France). The 3T MRI system (Prisma Fit, Siemens, Erlangen, Germany) guided the procedure, using the body coil as the transmit antenna and an 11 cm loop coil as the receiver antenna. The HIFU unit was controlled from the Thermoguide graphical user interface (version 1.37.2002, Image Guided Therapy, Pessac, France).

### MRI sequences

The gels were assessed with MR-ARFI and MRT to observe acoustic absorption. Simultaneous displacement and temperature were measured with MR-ARFI using a FLASH-ARFI-MRT sequence. Additionally, standard MRT was performed to ensure repeatability, to measure the acoustic absorption coefficient, to verify the focal point was in-plane, and to verify that the droplets were exhibiting more heating at higher concentrations. Care was taken to ensure that the positions of the MRT sonications and the MR-ARFI sonications were in distinct locations, as previous heating was indicated in the gels to affect the displacement measurements. The temperature and displacement map data were analyzed with a custom MATLAB script (The MathWorks, Inc., Natick, MA, USA).

To generate the FLASH-ARFI-MRT sequence, a standard fast low angle shot (FLASH) sequence (49) was modified to incorporate vertical motion encoding gradients (MEGs) in the focused ultrasound direction (7, 50, 51). The sequence featured symmetric bipolar MEGs centered near the external trigger, with equal amplitudes and opposite phase. The MEG duration was reduced to maintain echo time (TE) less than 15 ms for good signal-to-noise ratio (SNR). These tissue displacement measurements are relatively slow, with maximum displacement requiring a few milliseconds (8). Hence, the need to displace the external trigger from the center of the MEG gradients. A variable delay was incorporated to adjust the timing between the MEG pulse and an infrared fiber-optic signal to trigger the focused ultrasound with each repetition time (TR). The motion encoding gradients, which form the basis of the MR-ARFI imaging, were eventually incorporated into the standard Siemens MR thermometry EP_SEG_THERM sequence. This allowed for an EP_SEG_THERM_ARFI sequence, with the commercial sequence adjustments available, although the sequence was not used to generate the data in this report.

The phase contrast was equated to displacement and temperature from Equations 1 and 2 (7). The phase change in the displacement phase map can be increased linearly with a larger MEG gradient amplitude or longer MEG duration. Similarly, a larger TE will result in a larger phase contrast in the temperature phase images.


(1) $$\text{Δ}u_{i}=\frac{\left(\phi_{i}^{+}-\phi_{0}^{+}\right)-\left(\phi_{i}^{-}-\phi_{0}^{-}\right)}{2\gamma M\delta }$$

(2) $$\text{Δ}T_{i}=\frac{\left(\phi_{i}^{+}-\phi_{0}^{+}\right)+\left(\phi_{i}^{-}-\phi_{0}^{-}\right)}{2\gamma \chi TE\delta B_{0}}$$

Here, *u_i_* is pixel displacement (µm), *T_i_* is pixel temperature (°C), *γ* is gyromagnetic ratio of hydrogen (267.52e6 rad.s^-1^.T^-1^), *δ* is the MEG duration per lobe (ms), *M* is MEG gradient amplitude (mT.m^-1^), $\phi_{i}^{+}\;$ is the positive polarity pixel value during HIFU sonication, $\phi_{0}^{+}\;$ is the baseline positive polarity pixel value without HIFU sonication, $\phi_{i}^{-}\;$ is the negative polarity pixel value during HIFU sonication, $\phi_{0}^{-}\;$ is the baseline negative polarity pixel value without HIFU sonication, *B*_0_ is the main magnetic field magnitude of 3 T, *χ* is the proton resonance frequency shift (PRFS) temperature coefficient of -0.0094 ppm.°C^-1^.

For standard gradient recall echo (GRE) thermometry sequences, the optimal SNR in temperature phase imaging occurs with TE at the effective spin-spin relaxation time $\left(T_{2}^{*}\right)$ . (4) Since the FLASH-ARFI-MRT sequence is diffusion-weighted, the SNR is dependent on the MEG b-value. The minimum standard deviation of the average displacement measurement for the bipolar FLASH-ARFI-MRT sequence occurs approximately when one MEG lobe duration (*δ*) is equal to ½ $T_{2}^{*}$ . The b-value was determined from Equation 3 (7, 51).


(3) $$b=\frac{2}{3}{\left(\gamma M\right)}^{2}\delta^{3}$$

Where b is the b-value, *γ* is gyromagnetic ratio of hydrogen, *M* is MEG gradient amplitude, *δ* is the MEG duration per lobe.

The phantom structure and droplet viability were verified by a *T*_1_-VIBE sequence and by good repeatability with MR temperature measurements of standard HIFU sonication. These measures indicated that the droplets had not aggregated or undergone Ostwald ripening, and the TMM phantoms were fabricated adequately and were free of entrapped gas bubbles. Previous studies showed that repeated sonications on this droplet formula in TMM gel phantoms resulted in a slight decrease in size and almost identical temperature gains (41).

In the scanner, the gel position was determined with a “localizer”. A 3D *T*_1_-VIBE sequence was obtained for higher resolution and accuracy, for synchronizing the MRI coordinate position, verifying no prefocal abnormalities, and to place the specimen directly at the focal length of the HIFU transducer. The *T*_1_-VIBE sequence generally used parameters similar to the following: [TE, 1.81 ms; TR, 5.44 ms; acquisition time (TA), 4:09 m:s; flip angle (FA), 10°; bandwidth (BW), 390 Hz; echo planar imaging factor (EPI), 1; partial Fourier factor (PF), 1; slice thickness (SL), 1 mm; number of acquisitions (NA), 1; Field of View (FOV), 256 mm x 256 mm; acquisition matrix (AM), 320x320].

Standard MRT (EP_SEG_THERM, Siemens, Erlangen, Germany) was used for high-duty cycle and higher-temperature sonications, more similar to ablative conditions. The sequence used a multislice temperature sensitive RF-spoiled segmented GRE-EPI sequence composed of three interleaved slices to monitor temperature elevation: [TE, 8.62 ms; TR, 41.47 ms; TA, 2.12 s; FA, 20°; BW, 815 Hz; EPI, 1; PF, 1; SL, 5 mm; NA, 1; FOV, 128 mm x 128 mm; AM, 128x128]. Routine MRT sonication conditions were: [heating time (*T_H_*), 33 s; acoustic power (P), 48 W; duty cycle (DC), 90%; energy emitted per sonication, 1.43 kJ; acoustic frequency (f), 1.031 MHz].

The FLASH-ARFI-MRT sequence was used with low-duty cycle sonications, under conditions similar to mild hyperthermia for focused ultrasound-mediated chemotherapy delivery. The parameters varied slightly, but used values similar to the following: coronal view, [TE, 13 ms; TR, 150 ms; TA, 19.05 s; FA, 12°; BW, 120 Hz; EPI, 1; PF, 1; SL, 4 mm; NA, 1; FOV, 128 mm x 128 mm; AM, 128x128; fat suppression (FS), on; time shift from HIFU trigger to center of bipolar MEG lobes, -2 ms; *δ*, 4 ms; M, 30 mT.m^-1^; MEG ramp-up time, 300 µs; delay between end of slice select gradients to beginning of MEG lobes (*τ*), 200 µs]. The MR-ARFI sonications were triggered by the MRI unit and sonications occurred with each repetition time: [Time On, 7 ms; P, 67 W; f, 1.031 MHz]. This sequence performs a 7 ms sonication during each TR, giving a 4.67% DC. The b-value was determined from Equation 3 to be about 2.75 s.mm^-2^.

A relaxometry mapping protocol (Myomaps, Siemens, Erlangen, Germany) was created for spin-lattice relaxation time (*T*_1_) mapping, effective spin-lattice relaxation time ( $T_{1}^{*}$ ) mapping, and spin-spin relaxation time (*T*_2_) mapping. The effective spin-spin relaxation time ( $T_{2}^{*}$ ) should be measured in future studies to optimize the SNR of the FLASH-ARFI-MRT sequence. Susceptibility-weighted imaging (SWI) was added to the protocol. A 16-slice coronal SWI sequence used the following parameters: [TE, 20 ms; TR, 27 ms; FA, 15°; BW, 120 Hz; SL, 1 mm; NA, 1; FOV, 120 mm x 120 mm; AM, 128x128]. The protocol was performed on select lecithin-PFOB and F_8_TAC_18_-PFOB gel sets. The body coil was used as the transmit coil and the 11 cm loop coil as the receiver. Relaxometry and SWI map values were compared across selected gel sets, at all concentrations. The values were post-processed using an average region of interest in the gel centers, across multiple slices.

### Phantom and droplet absorption

From the MRT data, the change from baseline in the acoustic absorption coefficient at each droplet concentration interval was assessed. These values were measured relative to baseline, represented by the average of the control measurements in each individual gel set. A mono-exponential fit line was fit to the MRT data points using Equation 4.


(4) $$\alpha \left(\left[C\right]\right)=A\left(1-e^{\frac{-\left[C\right]}{C_{0}}}\right)$$

Where *α* is the acoustic absorption coefficient (m^-1^), *A* is the maximum change in the acoustic absorption coefficient (m^-1^), *C*_0_ is the critical concentration (% v:v), [C] is the percent volume fraction of the emulsion (% v:v).

The displacement data was compared between experiments, also relative to baseline. Denoted as the spatial integral displacement, all pixels with a measurable change in displacement values at the focal region were summed. The MR-ARFI spatial integral displacement data were fit with linear regression, and the fit line formulas along with R^2^ values were determined. The interaction radii for these sets of F_8_TAC_18_-PFOB emulsions were estimated from previous experiments using Equation 5 from *Holman et al.* (43)


(5) $$\frac{R_{int}}{R_{0}}\sqrt[{3}]{C_{0}}=\frac{{R^{\prime }}_{int}}{{R^{\prime }}_{0}}\sqrt[{3}]{{C^{\prime }}_{0}}$$

Where *R_int_* is the interaction radius (µm), *R*_0_ is the physical droplet radius (µm), *C*_0_ is the critical concentration (v:v). The prime symbol distinguishes the values from separate experiments. The mean interparticle distance at the critical concentration was determined by Equation 6 (52).


(6) $$a\left(C_{o}\right)=2R_{0}\left({\left(\frac{0.59}{C_{o}}\right)}^{1/3}-1\right)$$

Where *a*(*C*_0_) is the mean interparticle distance at the critical concentration (µm).

ECAH (Epstein-Carhart-Allegra-Hawley) theory was used to estimate the contribution of thermoviscous attenuation to acoustic absorption (53, 54). The formula is given in Equation 7 (55, 56).


(7) $$\frac{\alpha_{tv}}{\left[C\right]}=\frac{\frac{18\omega }{v_{m}}{\left(1-\frac{\rho_{m}}{\rho_{e}}\right)}^{2}\left(Y+1\right)Y^{2}}{4Y^{4}{\left(\frac{\rho_{m}}{\rho_{e}}+2\right)}^{2}+36Y^{3}\left(\frac{\rho_{m}}{\rho_{e}}+2\right)+162Y\left(Y+1\right){\left(\frac{\rho_{m}}{\rho_{e}}\right)}^{2}+81{\left(\frac{\rho_{m}}{\rho_{e}}\right)}^{2}}$$

The value Y is related to the skin depth and particle radius of the shear wave, given as $Y=R_{o}\sqrt{\frac{\omega }{2v_{m}}}$ . The variable *R*_o_ denotes the droplet radius (m), *α_tv_* is the thermoviscous attenuation (m^-1^), ω is the angular frequency (2*πf*, rad.s^-1^), *f* is the HIFU frequency (Hz), *v_m_* is the kinematic viscosity of the medium (m^2^.s^-1^), [*C*] is the droplet concentration (v:v), *ρ_e_* is the emulsion density (kg.m^-3^), and *ρ_m_* is the medium density (kg.m^-3^).

### Statistical analysis of acoustic absorption

The resulting data from MR thermometry and MR-ARFI integral displacement measurements were assessed statistically with a bootstrap method to determine if the surfactant groups were significantly different. The F_8_TAC_18_ and lecithin data sets were merged to a single set. Half the total data points were selected at random and collected in a group. A separate group was made with the remaining data points. Then, the two groups were separately fit with the same regression algorithm used previously, to obtain the critical concentration. For the bootstrap method, the difference between the critical concentrations was obtained at each iteration. This was repeated one thousand times to arrive at a histogram. The p-value was determined by finding the proportion of instances where the differences in the critical concentration were greater than those found in the original regression fitting.

### Hydrophone system

**Figure 3:**
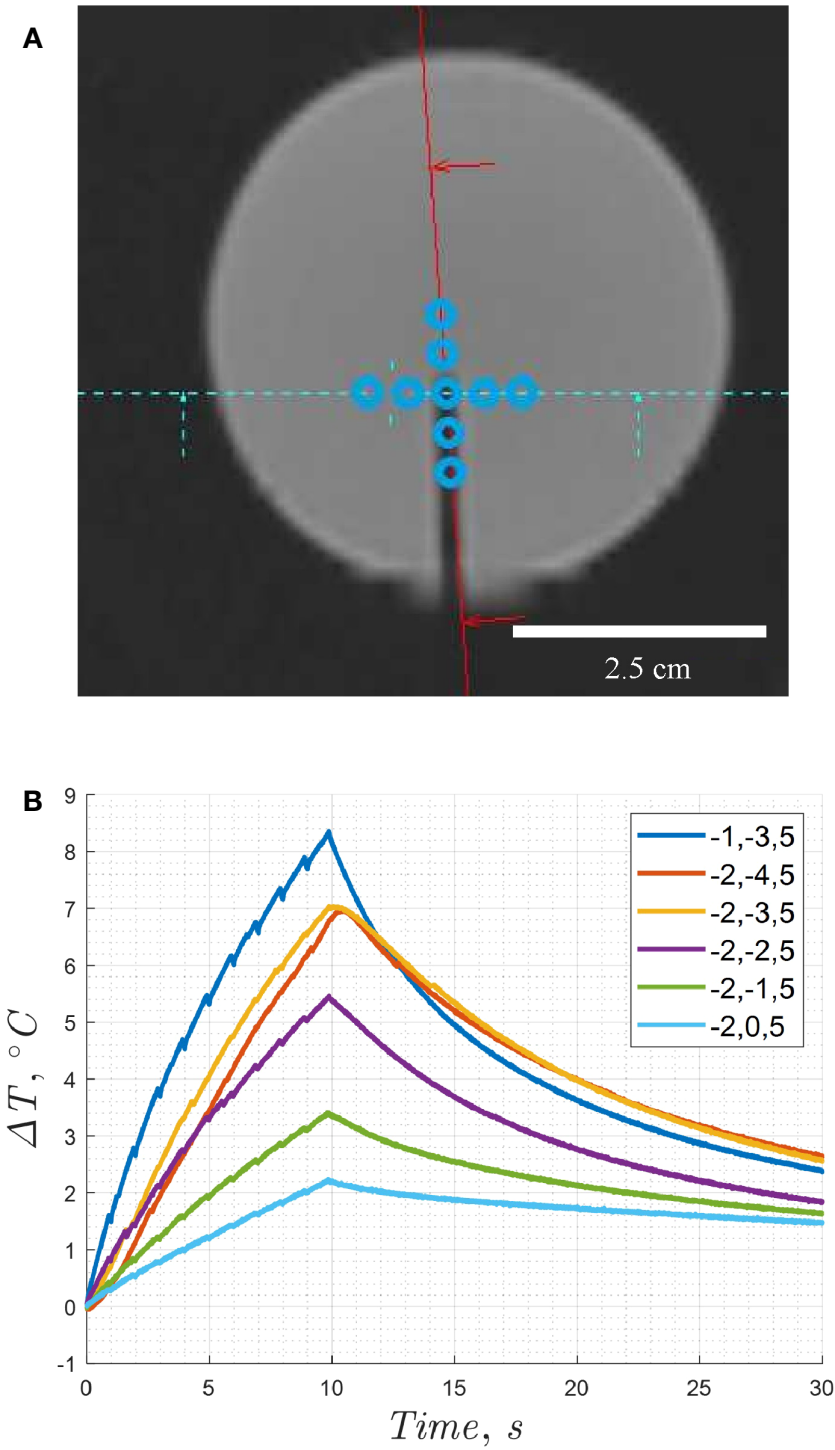
**(A)** Coronal view of MR image from a T1-VIBE sequence showing the position of the hydrophone needle within the tissue mimicking phantom sample. **(B)** Temperature increase compared to focal point coordinates, when controlled by electronic beam steering. Point (-1,-3,5) was chosen as the optimal location. Note, the estimated location of the focal spot relative to the sensor is within the distance of one voxel, providing a spatial precision of one-half voxel or 0.5 mm. The relative positions are enlarged to enhance visibility of the sonication pattern.

Fabry-Perot fiber-optic hydrophone (FOHS, Precision Acoustics, Dorchester, UK) measurements were performed on a control gel and a 0.3% v:w F_8_TAC_18_-PFOB gel. A catheter was used to guide a hydrophone needle to the gel center, shown in **Figure 3**. A custom fiber optic cable was provided by the manufacturer, which was longer than the standard size. The cable was long enough to reach the center of the MRI bore, while connected to the hydrophone which was located outside the MRI Faraday cage, within the control room. Damaging the sensor would have been costly and caused many delays in data acquisition, so an alternative configuration was chosen. The hydrophone was designed to be used parallel to the beam path, though a perpendicular configuration was adopted for these experiments to prevent damage. In previous studies, the directional sensitivity of the sensor varied with frequency and the decibel recordings increased by 10% (about 1 dB) to account for the altered directionality (57). The voltage waveform was equated to pressure, with a hydrophone fiber optic sensitivity value of 126.3 mV.MPa^-1^, corresponding to a HIFU frequency of 1.031 MHz, with uncertainties of 14% in this frequency range (57).

The actual hydrophone needle diameter was less than the MRI voxel resolution. The catheter size in **Figure 3A** appeared larger due to a susceptibility artifact. The focal spot was positioned incrementally, until the maximum temperature was obtained. The HIFU focal spot position was adjusted with electronic beam steering in the Thermoguide interface, as shown in **Figure 3**. First, the x-position was adjusted until the location for the maximum temperature increase was observed, and the coordinate was fixed. Next, the y-position was adjusted at this fixed x-coordinate until the maximum temperature increase was observed with equal sonication conditions. This location was then used for subsequent acoustic spectra recordings.

The hydrophone temperature recordings were performed before acoustic spectra recordings, to verify the samples were stable at increasing acoustic powers. The control gel was sonicated with a 99.9% duty cycle without simultaneous MRT, at 1.031 MHz, for 10 seconds with acoustic powers of 19 W, 32 W, 48 W, and 67 W. The 0.3% v:w perfluorocarbon gel was measured only at 67 W, with 5 second sonications to prevent overheating. The hydrophone voltage spectra were normalized to the main peak with logarithmic division. The relative increase in the harmonics between gels was compared to identify changes indicative of cavitation.

### NMR system and sequences

^19^F diffusion-ordered spectroscopy (DOSY) NMR was used to experimentally measure the droplet diffusion coefficient (58, 59). Topspin 4.0.5 (Bruker BioSpin Gmbh, Billerica, MA, USA) was used to obtain and process the spectra. The diffusion coefficient for a 2.3% v:v stock solution of 1.24 µm diameter F_8_TAC_18_-PFOB droplets was measured using a ^19^F-DOSY pulse sequence on an NMR system (300 MHz, Avance III HD NanoBay, Bruker, Billerica, MA, USA). The sequence was longitudinal eddy current delay (LED) with bipolar gradient pulses and two spoiler gradients (59, 60). The ^19^F-DOSY acquisition parameters included: [number of scans (NS), 16; size of FID (TD2 & TD1), 64k & 16; spectral width (SW), 200ppmx10ppm; relaxation delay (D1), 2 s; diffusion time (d20), 800 ms; gradient pulse (p30), 5000 µs; GAMMA, 4005.2 Hz.G^-1^]. The spectrum and sequence parameters are provided in the supplementary materials.

The theoretical Stokes-Einstein diffusion coefficient was estimated using Equation 8.


(8) $$D=\frac{k_{b}T}{6\pi \eta R_{o}}$$

Where the variable *D* denotes the diffusion coefficient of an individual PFOB droplet (m^2^.s^-1^), *k_b_* represents Boltzmann’s constant (J.K^-1^), *T* is the absolute temperature (K), *η* denotes the dynamic viscosity of the medium (Pa), and *R_o_* is the particle radius (m).

Then, the time-averaged diffusion distance during HIFU sonications were determined using Equation 9.


(9) $$<r^{2}>=6Dt$$

Here, t is time (s), r is the three-dimensional displacement distance (m), and D is the diffusion coefficient (m^2^.s^-1^).

Quantitative ^19^F-NMR was performed to verify the perfluorocarbon concentration in selected gel sets. The sequence was a 1D sequence with inverse gated decoupling and a 90° RF pulse (zgig), which generates a ^1^H-decoupled fluorine spectrum without the nuclear Overhouser effect (NOE). The quantitative NMR parameters included: [D1, 15 s; NS, 64; RG, 203; T, 298 K].

### Ultrasonography and perfusion system

The flow phantom used for ultrasonography was described in previous articles (42, 43). The system was perfused by a pulsatile perfusion machine that was originally designed to evaluate kidney function from non-heart beating organ donors, prior to transplant, with ^31^P-MRI spectroscopy (61). The perfusion system was composed of three primary components: the perfusion module, a drive module, and an “umbilical” system. The perfusion module was a container positioned within the MRI bore, in which was placed the kidney, or TMM phantom in this case. The drive module was composed of the hardware unit with a control interface, which was located in the MRI control room, and used compressed oxygen as the driver. An “umbilical” system connected the compressed oxygen to the perfusion module and the drive module, by passing through the ports in the Faraday cage.

To obtain the ultrasonography images, a specially designed 192-element MR-compatible abdominal ultrasound probe (IBMT, Fraunhofer, Sulzbach/Saar, Germany) was used with a clinical ultrasound scanner (Acuson Antares, Siemens Healthcare, Mountain View, CA; USA) to image cavitation effects during HIFU sonications. The transducer was designed to avoid interference from the MRI scanner during concurrent MRI and ultrasonography, with operating frequencies of 3-7.5 MHz and HIFU frequency at 1.031 MHz (37, 62, 63). The design reduced interference from the HIFU transducer and MRI system, allowing pulsed color Doppler and B-mode imaging of the perfused phantom.

### Rheometry analysis

Certain gel sets were characterized with rheometry measurements to estimate the elastic modulus, shear modulus, and viscosity. This was performed primarily in an attempt to relate the outlier displacement to the relative elasticity between the samples. Measurements were made on a HAAKE MARS 40 rheometer (Thermo Fisher Scientific, Waltham, MA, USA), with rotational frequencies between about 1-50 Hz. The recorded values included the complex shear modulus (G^*^), shear storage modulus (G’), shear loss modulus (G^”^), and the frequency (Hz). From this data, assuming a Poisson ratio of 0.5, the complex elastic modulus (E^*^) was estimated as 3G^*^. The dynamic viscosity was determined from Equation 10 (64). The viscosity value at 1 Hz frequency was used to approximate the zero-shear viscosity limit.


(10) $$\eta \;{\;}^{'}=\frac{G^{\;\;''}}{2\pi f}$$

## Results and discussion

### MRI displacement and temperature measurements

**Figure 4:**
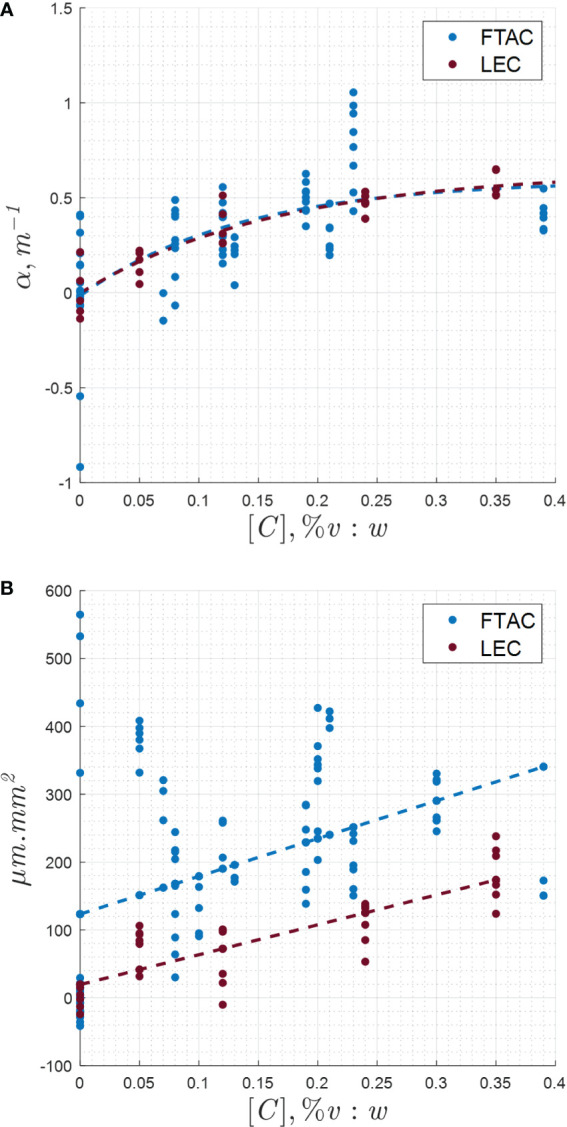
**(A)** Change in acoustic absorption coefficient with emulsion concentration in the TMM phantoms, for both the lecithin and F_8_TAC_18_ surfactants. **(B)** MR-ARFI displacement response with varying emulsion concentration and surfactant.

In **Figure 4A**, the sustained sonications, used with the MR-ARFI TMM phantoms, were fit with a mono-exponential function given in Equation 4. One data set was omitted from the figure due to droplet size differences. The regression line fit variables of the F_8_TAC_18_-PFOB measurements (n=74) and the lecithin-PFOB measurements (n=25) are given in **Table 1**, together with the estimation of the thermoviscous attenuation and interaction radius. Each MRT data point represents independent cycles of prolonged heating for 33 seconds followed by cooling to room temperature. From each cycle, a single measure of acoustic absorption was obtained. The data consisted of four sets of F_8_TAC_18_-PFOB measurements and two sets of lecithin-PFOB measurements, with about five gels per set, and about four to five measurements per gel (maximum of seven).

**Table 1:** Fit constants from equation 4 along with confidence intervals (95% CI).

Surfactant	*R*, (*µm*)	*C*_0_ (95% CI), (%v:v)	*A* (95% CI), (*m* ^-1^)	R^2^	*α_tv_* (*C*_0_), (*m* ^-1^)	*R_int_*, (µm)
F_8_TAC_18_	0.851-0.880	0.13 (0.01-0.26)	0.61 (0.35-0.87)	0.41	0.21	9.25-9.57
Lecithin	0.914	0.17 (0.05-0.28)	0.64 (0.47-0.81)	0.86	0.27	9.09

Also given is the estimated interaction radius and thermoviscous attenuation at the critical concentration.

In **Figure 4B**, the MR-ARFI spatial integral displacement data are shown. Outliers were observed in some scans. The outliers were possibly due to problems in constructing the samples for the individual experiment or technical difficulties with the MRgHIFU system. Simple linear regression was performed. The fit constants and R^2^ values are shown in **Table 2**.

**Table 2:** MR-ARFI simple linear regression fitting along with confidence intervals (95% CI) of the data points in **Figure 4B**.

Surfactant	Slope (95% CI)	Intercept (95% CI)	R^2^
F_8_TAC_18_	556.8 (295.5-818.1)	123.3 (80.79-165.8)	0.17
Lecithin	440.6 (320.1-561.1)	19.4 (-4.5-43.34)	0.67

The phantoms were effective for low-duty cycle MR-ARFI measurements between 40-45 °C, which were similar to mild hyperthermia applications, and sustained high-duty cycle MRT measurements, which were more similar to ablative sonication conditions. Even with low-energy and low-duty cycle sonications from MR-ARFI, the sonosensitive droplets were shown to increase ultrasound absorption. **Figure 4** shows an expected increasing trend in MRT temperature and MR-ARFI displacement as the droplet concentration increased. The spatial integral of the displacement field gave less noisy data, when compared to values of maximum displacement and temperature measurements. The values of maximum displacement, maximum temperature, and integral temperature were also obtained. However, these values did not tend to return to equilibrium before subsequent sonications and were omitted for brevity.

### Bootstrap statistics

Lecithin-PFOB droplets were previously tested extensively as intravenous radiological contrast agents, with results illustrating safety and efficacy in human subjects (65, 66). This F_8_TAC_18_-PFOB emulsion has not been assessed in human studies, but many aspects of the behaviour in humans can be anticipated by comparing to lecithin-PFOB clinical studies. A comparative measure was wanted using an alternative surfactant formula to identify any effects that could result from the surfactant alone. The lecithin-PFOB emulsion was chosen as a comparison for this reason. Similar cavitation effects from the two formulas, using droplets of equal sizes, should give similar measures of acoustic absorption. The comparison was performed to verify this experimentally. The results were assessed statistically. A bootstrap method was applied to the MR thermometry critical concentrations in **Figure 4A** and the slope of the MR-ARFI integral displacement data in **Figure 4B**. The results indicated that for both the MR thermometry data (p=0.38) and the MR-ARFI integral displacement data (p=0.34) in **Figure 4**, there was insufficient evidence to reject the null hypothesis. That is, there was no significant difference in the observations between the two surfactant groups.

### Hydrophone temperature and acoustic spectra measurements

**Figure 5:**
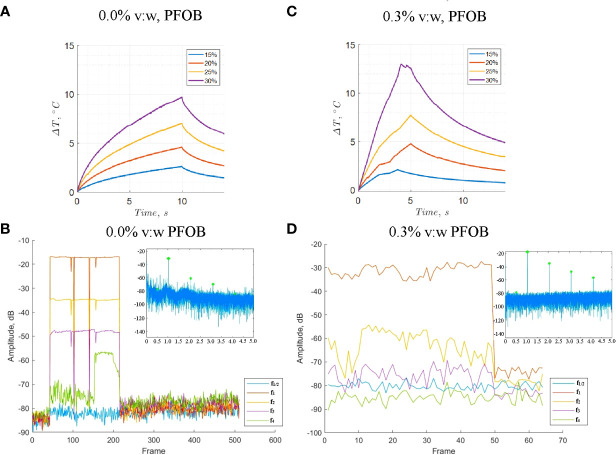
Hydrophone temperature measurements and change in acoustic spectra harmonics with time. **(A)** Temperature measurements in a control gel phantom. The measurements at 67 W were repeated three times. **(B)** Harmonic traces of control phantom with 0.0% v:w emulsion at 67 W. **(C)** Temperature measurements in a 0.3% v:w F_8_TAC_18_-PFOB TMM phantom. **(D)** Harmonic traces of phantom with 0.3% v:w F_8_TAC_18_-PFOB TMM phantom at 67 W.

The hydrophone temperature recordings in **Figures 5A, B** showed elevated temperatures in the perfluorocarbon sample compared to the control. The 0.3% v:w sample was heated at only half the duration of the control sample, though still showed an increased temperature profile. There was also a higher rate of change of temperature with time at 67 W, which may be due to nonlinear effects increasing the acoustic absorption coefficient. Techniques such as cavitation threshold measurements and passive acoustic mapping might be useful to quantify cavitation and nonlinear effects under a variety of scenarios, such as higher powers and alternate sonication conditions (39).

The hydrophone voltage recordings of the acoustic spectra are shown in **Figures 5C, D**. The 0.3% v:w F_8_TAC_18_-PFOB TMM gels showed lower amplitude sub-harmonics and increased ultra-harmonics, which might have contributed to the enhanced thermal absorption. **Table 3** gives the harmonic values of the hydrophone voltage spectra normalized to the main peak. For the 0.3% PFOB sample, the scans were performed at 67 W acoustic power, had a 1.6 MPa peak negative pressure, a 2.7 MPa peak-to-peak pressure, a mechanical index of 1.6, and a duty cycle of 4.7%. For the control gel, the acoustic power was 67 W, a 0.5 MPa peak negative pressure, a peak-to-peak pressure of 1.0 MPa, a mechanical index of 0.5, and a duty cycle of 4.7%. The spectra were noticeably different at higher HIFU amplitudes and in the perfluorocarbon sample. Normalized relative to the main peak, the subharmonics decreased by approximately a factor of 4 and the ultraharmonics increased by approximately a factor of 3, for the 0.3% v:w F_8_TAC_18_-PFOB sample compared to the control. Stable cavitation generally results in increased sub-harmonics and ultra-harmonics, while inertial cavitation increases broadband emissions, often resulting from collapsing microbubbles (67). As the acoustic absorption coefficient scales with frequency, higher frequency harmonics from cavitation and nonlinear wave propagation can result in increased acoustic absorption coefficients, resulting in increased heating (67).

**Table 3:** 1.031 MHz focused ultrasound harmonics at varying concentrations (% v:w) and focused ultrasound amplitudes, with harmonics normalized to the main frequency.

Concentration, [C] (% v:w)	Acoustic Power, P (W)	1/2 Harmonic, f_1/2_ (A.U.)	Main Harmonic, f_1_ (A.U.)	2nd Harmonic, f_2_ (A.U.)	3rd Harmonic, f_3_, (A.U.)	4th Harmonic, f_4_ (A.U.)	Summation, Σ (A.U.)
0.3	67	1.12e-3	1.00	1.33e-1	2.79e-2	1.22e-2	1.17
0.3	67	1.10e-3	1.00	1.32e-1	2.72e-2	1.04e-2	1.17
0.3	67	1.14e-3	1.00	1.33e-1	3.05e-2	1.04e-2	1.18
0.0	67	7.41e-3	1.00	5.69e-2	1.15e-2	9.33e-4	1.08
0.0	67	4.57e-3	1.00	5.07e-2	1.45e-2	2.16e-3	1.07
0.0	67	4.73e-3	1.00	5.75e-2	5.01e-3	3.72e-3	1.07
0.0	67	3.47e-3	1.00	4.52e-2	8.13e-3	2.79e-3	1.06

f_n_ distinguishes the harmonic frequency.

It is expected to be unfavorable for these droplets to undergo a phase change at these temperatures, acoustic pressures, and sonication conditions. Studies for low pressure chemotherapeutic drug delivery also illustrated a lack of PFOB vaporization under similar experimental conditions and droplet formula (20, 68). Droplets made with PFOB have generally been stable at larger pressures and have not been readily used for techniques such as acoustic droplet vaporization. In some instances, the droplets in this study appeared to produce cavitation at lower mechanical index than the FDA diagnostic ultrasonography limit of 1.9. At similar ultrasound frequencies, 233 nm perfluorohexane droplets, as adjuvants to histotripsy, have shown larger cavitation thresholds between 4-11 MPa, for a 0.35-3 MHz pulse frequency range (69). While Sonovue microbubbles have shown cavitation thresholds with peak refractory pressure of 0.25 MPa for pulse duration as low as 20 µs (70).

### SWI, relaxometry, and ultrasonography

**Figure 6:**
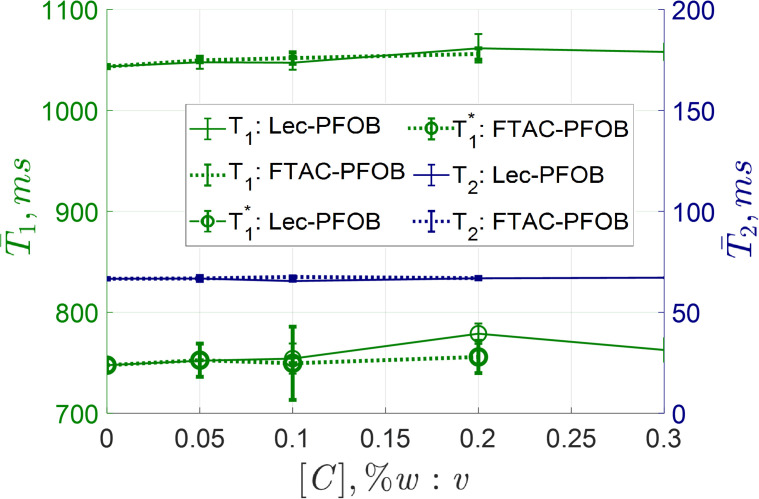
Average relaxometry mapping values across multiple coronal slices as a function of emulsion concentration. No noticeable trend was observed with increasing emulsion concentration. The T_1_ and T ^∗^_1_ mapping in the final F_8_TAC_18_ concentration was removed as the sample appeared to have possibly experienced cavitation during hydrophone recordings.

SWI was constant for all gel concentrations, with no definitive trend correlating with the emulsion concentration, except in the sample that possibly experienced cavitation. ^1^H-MRI relaxometry mapping of the water molecules in the TMM samples showed the *T*_1_, $T_{1}^{*}$ , and *T*_2_ were not affected by the surfactant and no measurable distinction in relaxometry due to possible variations in dissolved atmospheric gases. The relaxometry mapping values are given in **Figure 6**.

**Figure 7:**
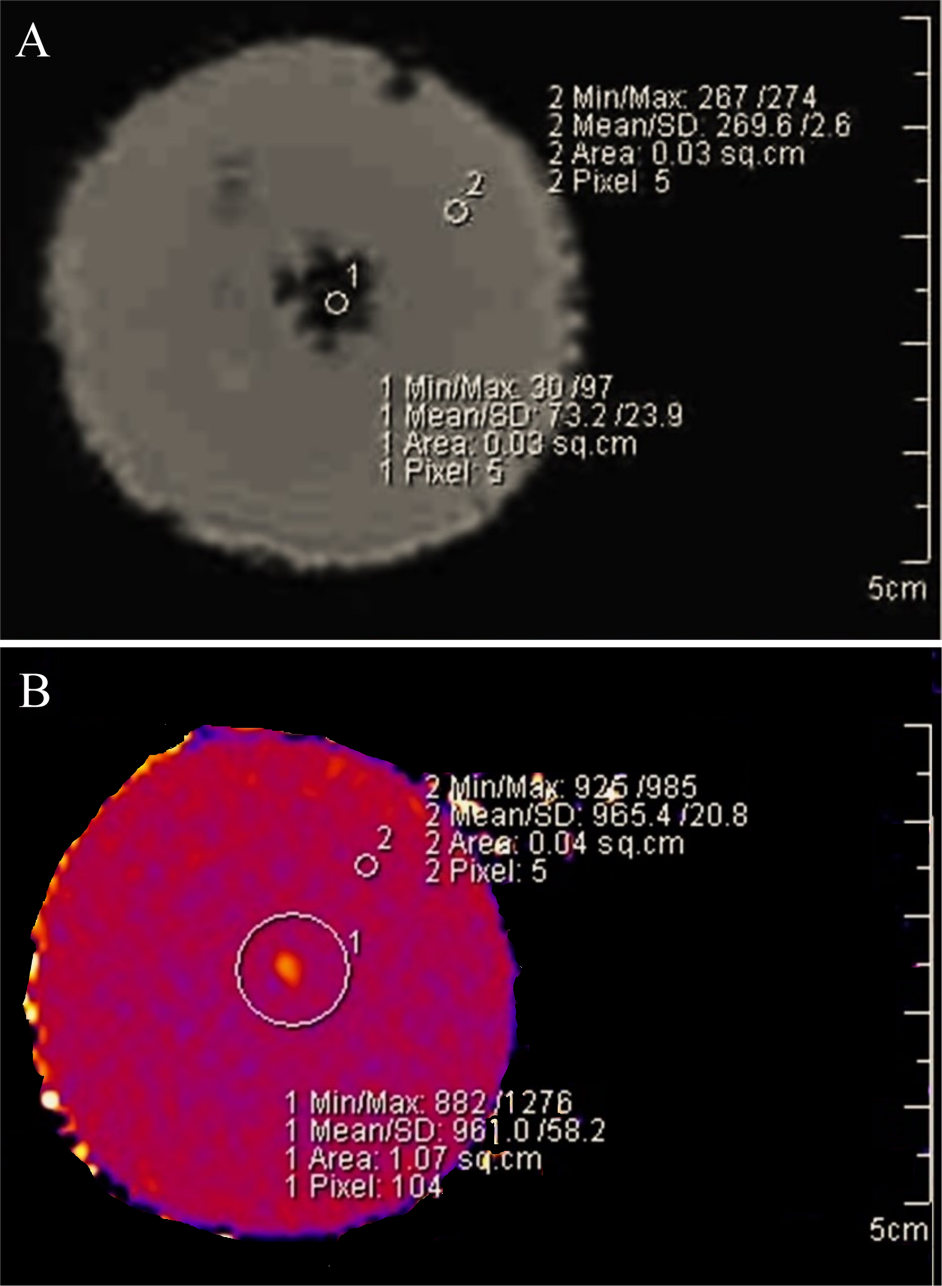
Susceptibility-weighted imaging and T_1_ relaxometry mapping. A residual bubble cloud appears to develop after HIFU sonications and likely contributes to the heat enhancement. **(A)** 0.3% v:w F_8_TAC_18_-PFOB gel phantom about one hour after HIFU heating during hydrophone recordings. The susceptibility effect was not seen in the control samples and disappeared within one week from the F_8_TAC_18_-PFOB sample. These measurements were performed after HIFU heating during hydrophone spectra recordings. **(B)** T_1_ hyperintensity was visible with relaxometry mapping.

Upon SWI and relaxometry imaging, the perfluorocarbon sample used during hydrophone measurements appeared to have undergone a phase change event during heating. The SWI and relaxometry maps indicated gas accumulation as shown in **Figures 7A, B**. A lesion was visible on these relaxometry maps, and a large hypointense signal was visible on SWI imaging, indicating likely gas accumulation at this location. Note that the SWI region is larger than the actual size of the gas volume, which is a result of a susceptibility artifact in the image. This artifact corresponded with a lesion in the same location, giving hyperintensity with *T*_1_, $T_{1}^{*}$ , and *T*_2_ mapping. The perfluorocarbon gel was measured one week later with the same sequence parameters and the hypointense SWI region had disappeared, indicating the gas accumulation had diffused back into the gel matrix.

**Figure 8:**
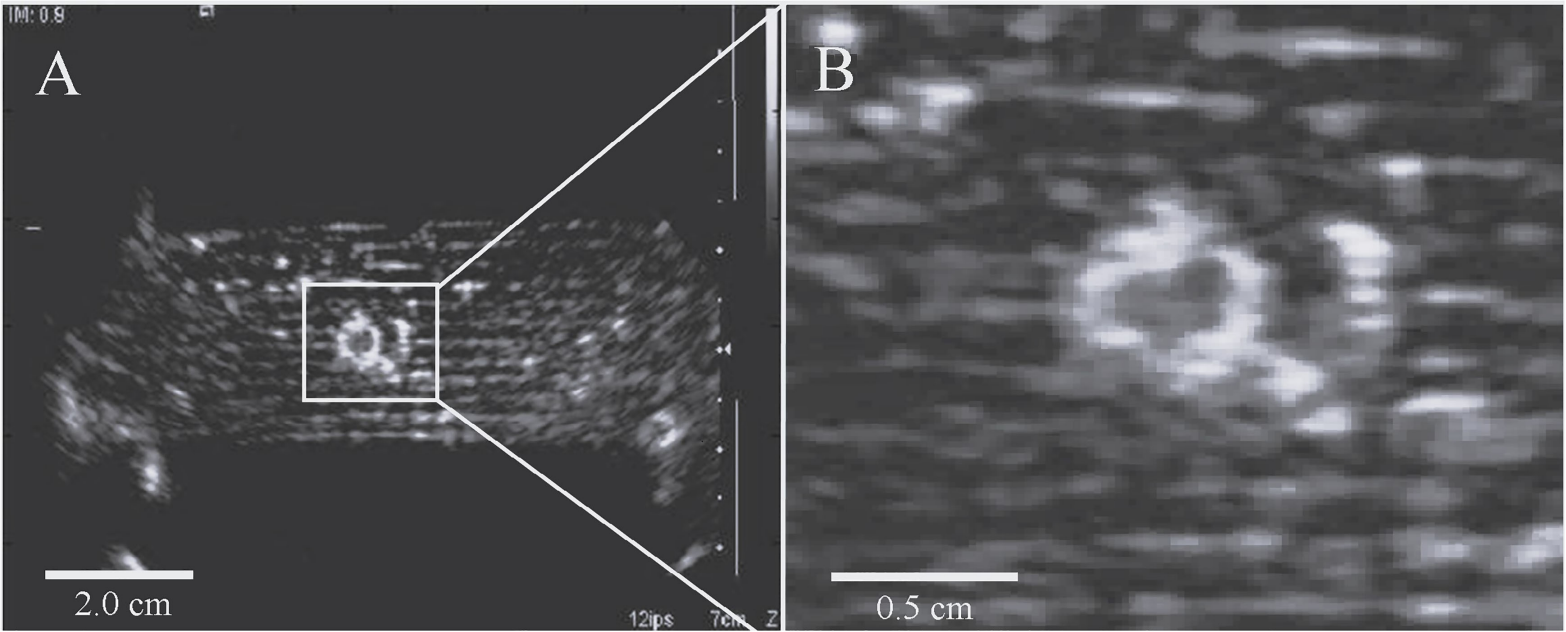
**(A)** Residual signal hyperintensity observed with B-mode ultrasonography after circular-pattern focused ultrasound sonication in perfused tissue mimicking phantom. **(B)** Magnified region of interest.

These findings corresponded to previously observed boiling core effects in *ex vivo* pig kidneys perfused with 0.24% v:v of this emulsion (42). Further observations of these effects were seen with MR-compatible ultrasonography, which illustrated residual hyperintensity after circular-patterned HIFU sonications, as shown in **Figures 8A**, **B**. For the video, please see the supplementary materials. In this case, these effects might be explained by sonosensitive droplets acting as cavitation nuclei, to reduce the pressure threshold needed to generate bubble clouds, as seen in boiling histotripsy (71). That is, with a distinct cavitation mechanism occurring at the droplet periphery, rather than within the liquid perfluorocarbon (72).

Local perfluorocarbon concentration can be measured with 19F-MRI, though the acquisition times can be long and dedicated hardware is additionally required. The emulsion might further benefit from incorporating Fe3+ chelate ions and a chelating agent into the solution, to reduce the 19F T1 relaxation time, increase sensitivity, and reduce acquisition times. (73, 74) However, the corresponding T2 shortening might lead to inaccurate temperature measurements with 1H-MRT.

^1^H-MRI oxygen mapping techniques to measure the change in *R*_1_, often employing inversion recovery sequences, have been reported in clinical studies for observing the change in local oxygen content after administering respiratory oxygen (75–77). It was tested if these effects, which could be an indirect measure of local concentration, could be observed under routine test conditions. The relaxometry measurements are shown in **Figure 6**. Relaxometry and SWI mapping were independent of emulsifier and showed no measurable distinction in values at higher droplet concentration, in absence of cavitation. The mean relaxometry in the TMM samples were measured at 1045-1085 ms, 720-790 ms, and 65-67 ms for *T*_1_, $T_{1}^{*}$ , and *T*_2_, respectively. Relaxometry values of MR-ARFI TMM phantoms were close to previously reported similar TMM phantoms and to human cardiac tissue (48, 78).

### Rheometry measurements

**Figure 9:**
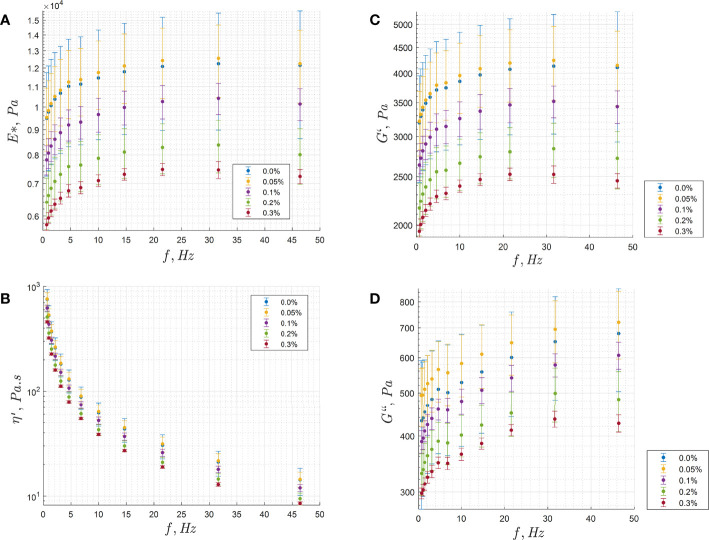
Rheometry analysis of a F8TAC18-PFOB TMM gel set. **(A)** Modulus of elasticity, **(B)** Dynamic viscosity, **(C)** Shear storage modulus, **(D)** Shear loss modulus.

The rheometry data is provided in **Figure 9**. The TMM phantoms tended to show a reduction in elastic modulus, shear modulus, and viscosity with increasing PFOB concentration. Dynamic viscosity showed a strong decrease with higher rotational frequency. The viscosity at 1 Hz of 450 Pa.s at 0.3% v:w was used to estimate the zero-shear viscosity, for determining the time-averaged diffusion distance of the droplets in the TMM sample. The TMM control phantom was measured to have an elastic modulus near 9.5 kPa. The elastic modulus was relatively stable across varying rotational frequencies, and was reduced with increasing perfluorocarbon concentration. Elastic modulus for porcine myocardium were reported at about 60 kPa (79), about 8 kPa in breast tissue (80), and 10-190 kPa for liver and kidney (80). Similar gel phantoms reported elastic modulus measurements between 8-34 kPa (47).

### ^19^F-DOSY NMR diffusion measurements

The Stokes-Einstein diffusion coefficient for 1.24 µm diameter particles in pure water at 298 K was determined to be 0.44 µm^2^.s^-1^ (81, 82). The ^19^F-DOSY measurement found a diffusion coefficient of 1.191 µm^2^.s^-1^, which was about 2.7 times larger than predicted by theory. This difference could be attributed to the emulsion solution having a lower dynamic viscosity than pure water. This corresponds with reduced viscosity at higher PFOB concentrations, as seen with rheometry measurements. An estimation was made for the time-averaged diffusion distance during HIFU sonications, accounting for size differences and medium viscosity, in both a previous parallel-strand TMM laminar flow model and these static ARFI samples. Comparison between time-averaged diffusion distance, the interaction radius, and the mean interparticle distance showed similar values, as shown in **Table 4**. In future studies, ^19^F-DOSY could allow changes in size distribution before and after HIFU, or assess samples from long-term storage (83).

**Table 4:** Comparison of theoretical diffusion distance, interparticle distance, interaction radius, and critical concentration across this study and previous studies.

Tissue Mimicking Model	Perfussion rate, _Wb._ (mL.s^-1^)	Droplet Radius *R*_0_, (µm)	Critical Conc, *C*_0_, (%v:w)	Interaction Radius, *R*_int_, (µm)	Time-averaged diffusion distance <*r*>, (µm)	Mean Interparticle Distance *a*(*C_0_*), (µm)
Laminar flow model (42)	0.1	1.15	0.13	12.5	11.3	13.4
F_8_TAC_18_-PFOB MR-ARFI model	0.0	0.88	0.13	9.57	0.02	13.4

### Interaction radius and diffusion analysis

It appeared that the short pulse duration of a few milliseconds for the MR-ARFI sequence generated a more linear temperature elevation with an increase in perfluorocarbon concentration. The longer HIFU pulse duration used in sustained sonication mode (tens of seconds) generated a nonlinear response that was fit with a first order asymptotic exponential function (43). This could be explained by the short pulse duration, that created a small interaction radius surrounding each droplet, which did not interact with neighboring droplets. However, for sustained sonications, the long pulses created an extended interaction radius surrounding each droplet, which increased beyond half the mean distance between particles and the heating effects saturated (43). Diffusion effects were not expected in MR-ARFI TMM due to the highly viscous gel structure. In free liquid solution, the diffusion distance appeared on the same order of magnitude as previous measurements of the interaction radius, as shown in **Table 4**. This suggested that the saturating effects might be influenced by diffusion in a perfused TMM flow phantom and perfused tissue.

Also, the interaction radius around an individual droplet was anticipated to be about 12.5 µm for a droplet that was 1.15 µm in radius, which would indicate increased acoustic absorption that results from a combination of thermoviscous heating and stable cavitation (43). The effects of stable cavitation were further supported by hydrophone spectra measurements observed in this current study. In a recent study, a novel cavitation mechanism was observed at negative pressures near 20 MPa, at the interface of unemulsified PFOB droplets in water, which could also influence the enhanced heating (72). That is, heterogeneous cavitation occurs at the external hydrophilic/hydrophobic surface of the droplets, forming cavitation bubbles consisting of water steam, and dissolved exogenous gases like oxygen and nitrogen. These cavitation bubbles can then further act as cavitation sites upon successive cycles from the incident HIFU pressure wave, by drawing more dissolved gases from the surrounding medium. Other studies showed nucleation occurring in the droplet interior when studying alternative perfluorocarbon emulsions for acoustic droplet vaporization, but the effects from this particular droplet formula appear to exhibit a distinct mechanism. Since the surrounding medium in this case is a TMM gel, with a high viscosity, the cavitation effects might be reduced because the diffusion rate of the individual droplet and dissolved exogenous gases is much lower in the highly viscous medium. This would correspond with previously observed effects of further enhancement of these droplets in free liquid solution, compared to the droplets embedded in TMM gels.

## Conclusions

In this study, a novel application using MR-ARFI for monitoring acoustic absorption from colloid-enhanced MRgHIFU therapy was assessed *in vitro*. Embedding TMM phantoms with perfluorocarbon emulsion adjuvants resulted in an increased focal spot displacement and a simultaneous temperature increase, with higher emulsion concentration. This increase in acoustic absorption and heating with a higher droplet concentration was also observed with sustained sonications, here and in previous studies (41–43). The change in acoustic absorption coefficient and integral displacement with droplet concentration was independent of surfactant, when comparing droplets of the same size. The results illustrated the great potential for the use of perfluorocarbon droplets to enhance magnetic resonance-guided thermal therapies. The techniques in this study could be applied to evaluate thermal enhancement with MRgFUS using a variety of colloids, including microbubbles, nanoparticles, and liposomes. A unique tissue mimicking gel phantom formula was developed that was compatible for use with both MR-ARFI and sustained MRgHIFU, with similar characteristics as *ex vivo* pig organs. Moreover, much insight was gained into the mechanism of enhanced acoustic absorption for this sonosensitive PFOB droplet formula. Further understanding could potentially help to improve ablative MRgHIFU procedures or even low power MRgFUS procedures like focused ultrasound-mediated drug delivery.

## Data availability statement

The raw data supporting the conclusions of this article will be made available by the authors, without undue reservation.

## Author contributions

All authors listed have made a substantial, direct, and intellectual contribution to the work, and approved it for publication.

## References

[1] Hindman JC. Proton resonance shift of water in the gas and liquid states. J Chem Phys (1966) 44(12):4582–92. doi: 10.1063/1.1726676

[2] Ishihara Y Calderon A Watanabe H Okamoto K Suzuki Y Kuroda K. A precise and fast temperature mapping using water proton chemical shift. Magn Reson Med (1995) 34(6):814–23. doi: 10.1002/mrm.1910340606 8598808

[3] Poorter JD Wagter CD Deene YD Thomsen C Ståhlberg F Achten E. Noninvasive mri thermometry with the proton resonance frequency (prf) method: in vivo results in human muscle. Magn Reson Med (1995) 33(1):74–81. doi: 10.1002/mrm.1910330111 7891538

[4] Rieke V Butts Pauly K. Mr Thermometry. J Magn Reson Imaging (2008) 27(2):376–90. doi: 10.1002/jmri.21265 PMC2780364 18219673

[5] Quesson B de Zwart JA Moonen CT. Magnetic resonance temperature imaging for guidance of thermotherapy. J Magn Reson Imaging (2000) 12(4):525–33. doi: 10.1002/1522-2586(200010)12:4<525::AID-JMRI3>3.0.CO;2-V 11042633

[6] Salomir R Hyacinthe J-N Viallon M. Method and apparatus for magnetic resonance guided high intensity focused ultrasound focusing under simultaneous temperature monitoring. U.S. Patent Nr. US 8,427,154 B2 (2013).

[7] Auboiroux V Viallon M Roland J Hyacinthe J Petrusca L Morel DR. Arfi-prepared mrghifu in liver: Simultaneous mapping of arfi-displacement and temperature elevation, using a fast gre-epi sequence. Magn Reson Med (2012) 68(3):932–46. doi: 10.1002/mrm.23309 22246646

[8] McDannold N Maier SE. Magnetic resonance acoustic radiation force imaging. Med Phys (2008) 35(8):3748–58. doi: 10.1118/1.2956712 PMC2673647 18777934

[9] Bitton RR Pauly KRB. Mr-Acoustic radiation force imaging (mr-arfi) and susceptibility weighted imaging (swi) to visualize calcifications in ex vivo swine brain. J Magn Reson Imaging (2014) 39(5):1294–300. doi: 10.1002/jmri.24255 PMC3983173 24123504

[10] Payne A Merrill R Minalga E Hadley JR Odeen H Hofstetter LW. A breast-specific mr guided focused ultrasound platform and treatment protocol: First-in-human technical evaluation. IEEE Trans BioMed Eng (2021) 68(3):893–904. doi: 10.1109/TBME.2020.3016206 32784128 PMC7878578

[11] Vappou J Bour P Marquet F Ozenne V Quesson B. Mr-Arfi-based method for the quantitative measurement of tissue elasticity: Application for monitoring hifu therapy. Phys Med Biol (2018) 63(9):095018. doi: 10.1088/1361-6560/aabd0d 29633958

[12] Bour P Marquet F Ozenne V Toupin S Dumont E Aubry J. Real-time monitoring of tissue displacement and temperature changes during mr-guided high intensity focused ultrasound. Magn Reson Med (2017) 78(5):1911–21. doi: 10.1002/mrm.26588 28090656

[13] Kaye EA Chen J Pauly KB. Rapid mr-arfi method for focal spot localization during focused ultrasound therapy. Magn Reson Med (2011) 65(3):738–43. doi: 10.1002/mrm.22662 PMC4099471 21337406

[14] Vyas U Kaye E Pauly KB. Transcranial phase aberration correction using beam simulations and mr-arfi. Med Phys (2014) 41(3):032901. doi: 10.1118/1.4865778 24593740 PMC3978249

[15] Ozenne V Constans C Bour P Santin MD Valabrègue R Ahnine H. Mri monitoring of temperature and displacement for transcranial focus ultrasound applications. NeuroImage (2020) 204:116236. doi: 10.1016/j.neuroimage.2019.116236 31597085

[16] Frenkel V Oberoi J Stone MJ Park M Deng C Wood BJ. Pulsed high-intensity focused ultrasound enhances thrombolysis in an in vitro model. Radiology (2006) 239(1):86–93. doi: 10.1148/radiol.2391042181 16493016 PMC2386885

[17] Landau LD Lifšic EM Lifshitz EM Kosevich AM Pitaevskii LP. Theory of elasticity: volume 7 Vol. 7. Amsterdam, Netherlands: Elsevier (1986).

[18] Lizzi FL Muratore R Deng CX Ketterling JA Alam SK Mikaelian S. Radiation-force technique to monitor lesions during ultrasonic therapy. Ultrasound Med Biol (2003) 29(11):1593–605. doi: 10.1016/S0301-5629(03)01052-4 14654155

[19] Payne A de Bever J Farrer A Coats B Parker DL Christensen DA. A simulation technique for 3d mr-guided acoustic radiation force imaging. Med Phys (2015) 42(2):674–84. doi: 10.1118/1.4905040 PMC4297281 25652481

[20] Al Rifai N Desgranges S Le Guillou-Buffello D Giron A Urbach W Nassereddine M. Ultrasound-triggered delivery of paclitaxel encapsulated in an emulsion at low acoustic pressures. J Mater Chem B (2020) 8:1640–8. doi: 10.1039/C9TB02493J 32011617

[21] Bérard C Desgranges S Dumas N Novell A Larrat B Hamimed M. Perfluorocarbon nanodroplets as potential nanocarriers for brain delivery assisted by focused ultrasound-mediated blood-brain barrier disruption. Pharmaceutics (2022) 14(7):1–25. doi: 10.3390/pharmaceutics14071498 PMC9323719 35890391

[22] Lyon PC Gray MD Mannaris C Folkes LK Stratford M Campo L. Safety and feasibility of ultrasound-triggered targeted drug delivery of doxorubicin from thermosensitive liposomes in liver tumours (tardox): A single-centre, open-label, phase 1 trial. Lancet Oncol (2018) 19(8):1027–39. doi: 10.1016/S1470-2045(18)30332-2 PMC6073884 30001990

[23] Gray MD Lyon PC Mannaris C Folkes LK Stratford M Campo L. Focused ultrasound hyperthermia for targeted drug release from thermosensitive liposomes: Results from a phase i trial. Radiology (2019) 291(1):232–8. doi: 10.1148/radiol.2018181445 30644817

[24] Holman R Lorton O Guillemin PC Peloso A Ricoeur A Salomir R. Magnetic resonance-guided focused ultrasound in the treatment of colorectal cancer liver metastases. In: Jeong KY, editor. Recent understanding of colorectal cancer treatment. Rijeka: IntechOpen (2022). doi: 10.5772/intechopen.105906

[25] Petrusca L Salomir R Manasseh G Becker CD Terraz S. Spatio-temporal quantitative thermography of pre-focal interactions between high intensity focused ultrasound and the rib cage. Int J Hyperthermia (2015) 31(4):421–32. doi: 10.3109/02656736.2015.1009501 25753370

[26] Zaccagna F Anzidei M Sandolo F Cavallo Marincola B Palla C Leonardi A. Mrgfus for liver and pancreas cancer treatments: the umberto i hospital experience. Transl Cancer Res (2014) 3(5):430–41. doi: 10.3978/j.issn.2218-676X.2014.09.03

[27] Okada A Murakami T Mikami K Onishi H Tanigawa N Marukawa T. A case of hepatocellular carcinoma treated by mr-guided focused ultrasound ablation with respiratory gating. Magn Reson Med Sci (2006) 5(3):167–71. doi: 10.2463/mrms.5.167 17139143

[28] Fischer K Gedroyc W Jolesz FA. Focused ultrasound as a local therapy for liver cancer. Cancer J (2010) 16(2):118–24. doi: 10.1097/PPO.0b013e3181db7c32 20404608

[29] Sehmbi AS Froghi S Oliveira de Andrade M Saffari N Fuller B Quaglia A. Systematic review of the role of high intensity focused ultrasound (hifu) in treating malignant lesions of the hepatobiliary system. HPB (2021) 23(2):187–96. doi: 10.1016/j.hpb.2020.06.013 32830069

[30] Jung SE Cho SH Jang JH Han JY. High-intensity focused ultrasound ablation in hepatic and pancreatic cancer: complications. Abdom Imaging (2011) 36(2):185–95. doi: 10.1007/s00261-010-9628-2 20512487

[31] Tsang SH Ma KW She WH Chu F Lau V Lam SW. High-intensity focused ultrasound ablation of liver tumors in difficult locations. Int J Hyperthermia (2021) 38(2):56–64. doi: 10.1080/02656736.2021.1933217 34420450

[32] Salomir R Petrusca L Auboiroux V Muller A Vargas MI Morel DR. Magnetic resonance-guided shielding of prefocal acoustic obstacles in focused ultrasound therapy: application to intercostal ablation in liver. Invest Radiol (2013) 48(6):366–80. doi: 10.1097/RLI.0b013e31827a90d7 23344514

[33] Lorton O Guillemin PC M’Rad Y Peloso A Boudabbous S Charbonnier C. A novel concept of a phased-array hifu transducer optimized for mr-guided hepatic ablation: Embodiment and first in-vivo studies. Front Oncol (2022) 12. doi: 10.3389/fonc.2022.899440 PMC9235567 35769711

[34] Preiswerk F De Luca V Arnold P Celicanin Z Petrusca L Tanner C. Model-guided respiratory organ motion prediction of the liver from 2d ultrasound. Med Image Anal (2014) 18(5):740–51. doi: 10.1016/j.media.2014.03.006 24835181

[35] Muller A Petrusca L Auboiroux V Valette PJ Salomir R Cotton F. Management of respiratory motion in extracorporeal high-intensity focused ultrasound treatment in upper abdominal organs: current status and perspectives. Cardiovasc Intervent Radiol (2013) 36(6):1464–76. doi: 10.1007/s00270-013-0713-0 24178235

[36] Mori N Jud C Salomir R Cattin PC. Leveraging respiratory organ motion for non-invasive tumor treatment devices: A feasibility study. Phys Med Biol (2016) 61(11):4247–67. doi: 10.1088/0031-9155/61/11/4247 27191374

[37] Lorton O Guillemin PC Möri N Crowe LA Boudabbous S Terraz S. Self-scanned hifu ablation of moving tissue using real-time hybrid us-mr imaging. IEEE Trans BioMed Eng (2018) 66(8):2182–91. doi: 10.1109/TBME.2018.2885233 30530308

[38] Bunevicius A McDannold NJ Golby AJ. Focused ultrasound strategies for brain tumor therapy. Oper Neurosurg (2020) 19(1):9–18. doi: 10.1093/ons/opz374 PMC7293897 31853548

[39] Stride E Coussios C. Nucleation, mapping and control of cavitation for drug delivery. Nat Rev Phys (2019) 1(8):495–509. doi: 10.1038/s42254-019-0074-y

[40] Astafyeva K Somaglino L Desgranges S Berti R Patinote C Langevin D. Perfluorocarbon nanodroplets stabilized by fluorinated surfactants: characterization and potentiality as theranostic agents. J Mater Chem B (2015) 3:2892–907. doi: 10.1039/C4TB01578A 32262418

[41] Desgranges S Lorton O Gui-Levy L Guillemin P Celicanin Z Hyacinthe JN. Micron-sized pfob liquid core droplets stabilized with tailored-made perfluorinated surfactants as a new class of endovascular sono-sensitizers for focused ultrasound thermotherapy. J Mater Chem B (2019) 7:927–39. doi: 10.1039/C8TB01491D 32255098

[42] Lorton O Guillemin PC Holman R Desgranges S Gui L Crowe LA. Enhancement of hifu thermal therapy in perfused tissue models using micron-sized ftac-stabilized pfob-core endovascular sonosensitizers. Int J Hyperthermia (2020) 37(1):1116–30. doi: 10.1080/02656736.2020.1817575 PMC8352380 32990101

[43] Holman R Gui L Lorton O Guillemin P Desgranges S Contino-Pépin C. Pfob sonosensitive microdroplets: Determining their interaction radii with focused ultrasound using mr thermometry and a gaussian convolution kernel computation. Int J Hyperthermia (2022) 39(1):108–19. doi: 10.1080/02656736.2021.2021304 35000497

[44] Contino-Pepin C Maurizis JC Pucci B. Amphiphilic oligomers: A new kind of macromolecular carrier of antimitotic drugs. Curr Med Chem Anticancer Agents (2002) 2(6):645–65. doi: 10.2174/1568011023353732 12678718

[45] Pucci B Maurizis J Pavia A. Telomeres et cotelomeres d’interet biologique et biomedical–iv. les telomeres du tris(hydroxymethyl)-acrylamidomethane nouveaux agents amphiphiles non ioniques. Eur Polym J (1991) 27(10):1101–6. doi: 10.1016/0014-3057(91)90087-5

[46] Lorton O Hyacinthe JN Desgranges S Gui L Klauser A Celicanin Z. Molecular oxygen loading in candidate theranostic droplets stabilized with biocompatible fluorinated surfactants: Particle size effect and application to *in situ* 19f mri mapping of oxygen partial pressure. J Magn Reson (2018) 295:27–37. doi: 10.1016/j.jmr.2018.07.019 30096550

[47] Farrer AI Odéen H de Bever J Coats B Parker DL Payne A. Characterization and evaluation of tissue-mimicking gelatin phantoms for use with mrgfus. J Ther Ultrasound (2015) 3(1):1–11. doi: 10.1186/s40349-015-0030-y 26146557 PMC4490606

[48] Hofstetter LW Fausett L Mueller A Odéen H Payne A Christensen DA. Development and characterization of a tissue mimicking psyllium husk gelatin phantom for ultrasound and magnetic resonance imaging. Int J Hyperthermia (2020) 37(1):283–90. doi: 10.1080/02656736.2020.1739345 PMC7748394 32204632

[49] Haase A Frahm J Matthaei D Hänicke W Merboldt KD. Flash imaging: rapid nmr imaging using low flip-angle pulses. 1986. J Magn Reson Imaging (2011) 213(2):533–41. doi: 10.1016/0022-2364(86)90433-6 22152368

[50] Souchon R Salomir R Beuf O Milot L Grenier D Lyonnet D. Transient mr elastography (t-mre) using ultrasound radiation force: theory, safety, and initial experiments *in vitro* . Magn Reson Med (2008) 60(4):871–81. doi: 10.1002/mrm.21718 18816871

[51] Chen J Watkins R Pauly KB. Optimization of encoding gradients for mr-arfi. Magn Reson Med (2010) 63(4):1050–8. doi: 10.1002/mrm.22299 PMC2852270 20373406

[52] Hao T Riman RE. Calculation of interparticle spacing in colloidal systems. J Colloid Interface Sci (2006) 297(1):374–7. doi: 10.1016/j.jcis.2004.10.014 16515794

[53] Epstein PS Carhart RR. The absorption of sound in suspensions and emulsions. i. water fog in air. J Acoust Soc Am (1953) 25(3):553–65. doi: 10.1121/1.1907107

[54] Allegra JR Hawley SA. Attenuation of sound in suspensions and emulsions: Theory and experiments. J Acoust Soc Am (1972) 51(5B):1545–64. doi: 10.1121/1.1912999

[55] Bera C Devarakonda SB Kumar V Ganguli AK Banerjee RK. The mechanism of nanoparticle-mediated enhanced energy transfer during high-intensity focused ultrasound sonication. Phys Chem Chem Phys (2017) 19(29):19075–82. doi: 10.1039/C7CP03542J 28702635

[56] Sadeghi-Goughari M Jeon S Kwon HJ. Analytical and numerical model of high intensity focused ultrasound enhanced with nanoparticles. IEEE Trans BioMed Eng (2020) 67(11):3083–93. doi: 10.1109/TBME.2020.2975746 32091987

[57] Morris P Hurrell A Shaw A Zhang E Beard P. A fabry–pérot fiber-optic ultrasonic hydrophone for the simultaneous measurement of temperature and acoustic pressure. J Acoust Soc Am (2009) 125(6):3611–22. doi: 10.1121/1.3117437 19507943

[58] Franconi F Lemaire L Gimel JC Bonnet S Saulnier P. Nmr diffusometry: A new perspective for nanomedicine exploration. J Control Release (2021) 337:155–67. doi: 10.1016/j.jconrel.2021.07.025 34280413

[59] Simeonova M Rangel M Ivanova G. Nmr study of the supramolecular structure of dual drug-loaded poly(butylcyanoacrylate) nanoparticles. Phys Chem Chem Phys (2013) 15:16657–64. doi: 10.1039/C3CP51471D 23970022

[60] Wu D Chen A Johnson CS. An improved diffusion-ordered spectroscopy experiment incorporating bipolar-gradient pulses. J Magn Reson Ser A (1995) 115(2):260–4. doi: 10.1006/jmra.1995.1176

[61] Buchs JB Bühler L Morel P. A new disposable perfusion machine, nuclear magnetic resonance compatible, to test the marginal organs and the kidneys from non-heart-beating donors before transplantation. Interact Cardiovasc Thorac Surg (2007) 6(4):421–4. doi: 10.1510/icvts.2006.146043 17669888

[62] Crowe LA Manasseh G Chmielewski A Hachulla AL Speicher D Greiser A. Spatially resolved mr-compatible doppler ultrasound: Proof of concept for triggering of diagnostic quality cardiovascular mri for function and flow quantification at 3t. IEEE Trans BioMed Eng (2018) 65(2):294–306. doi: 10.1109/TBME.2017.2764111 29053451

[63] Santini F Gui L Lorton O Guillemin PC Manasseh G Roth M. Ultrasound-driven cardiac mri. Phys Med (2020) 70:161–8. doi: 10.1016/j.ejmp.2020.01.008 32032800

[64] Barnes HA Hutton JF Walters K. An introduction to rheology Vol. 3. . Amsterdam, Netherlands: Elsevier (1989).

[65] Holman R Lorton O Guillemin PC Desgranges S Contino-Pépin C Salomir R. Perfluorocarbon emulsion contrast agents: A mini review. Front Chem (2022) 9:1169. doi: 10.3389/fchem.2021.810029 PMC8785234 35083198

[66] Bruneton JN Falewée MN François E Cambon P Philip C Riess JG. Liver, spleen, and vessels: preliminary clinical results of ct with perfluorooctylbromide. Radiology (1989) 170(1):179–83. doi: 10.1148/radiology.170.1.2909093 2909093

[67] ter Haar G Coussios C. High intensity focused ultrasound: Physical principles and devices. Int J Hyperthermia (2007) 23(2):89–104. doi: 10.1080/02656730601186138 17578335

[68] Aliabouzar M Kumar KN Sarkar K. Effects of droplet size and perfluorocarbon boiling point on the frequency dependence of acoustic vaporization threshold. J Acoust Soc Am (2019) 145(2):1105–16. doi: 10.1121/1.5091781 PMC7112712 30823782

[69] Vlaisavljevich E Aydin O Lin KW Durmaz YY Fowlkes B ElSayed M. The role of positive and negative pressure on cavitation nucleation in nanodroplet-mediated histotripsy. Phys Med Biol (2015) 61(2):663–82. doi: 10.1088/0031-9155/61/2/663 26716568

[70] Lin Y Lin L Cheng M Jin L Du L Han T. Effect of acoustic parameters on the cavitation behavior of sonovue microbubbles induced by pulsed ultrasound. Ultrason Sonochem (2017) 35:176–84. doi: 10.1016/j.ultsonch.2016.09.016 27707644

[71] Xu Z Hall TL Vlaisavljevich E Lee FT Jr. Histotripsy: the first noninvasive, non-ionizing, non-thermal ablation technique based on ultrasound. Int J Hyperthermia (2021) 38(1):561–75. doi: 10.1080/02656736.2021.1905189 PMC9404673 33827375

[72] Pfeiffer P Shahrooz M Tortora M Casciola CM Holman R Salomir R. Heterogeneous cavitation from atomically smooth liquid–liquid interfaces. Nat Phys (2022), 18:1431–5. doi: 10.1038/s41567-022-01764-z

[73] Jahromi AH Wang C Adams SR Zhu W Narsinh K Xu H. Fluorous-soluble metal chelate for sensitive fluorine-19 magnetic resonance imaging nanoemulsion probes. ACS Nano (2019), 143–51. doi: 10.1021/acsnano.8b04881 PMC6467752 30525446

[74] Kislukhin AA Xu H Adams SR Narsinh KH Tsien RY Ahrens ET. Paramagnetic fluorinated nanoemulsions for sensitive cellular fluorine-19 magnetic resonance imaging. Nat Mater (2016), 662–8. doi: 10.1038/nmat4585 PMC5053764 26974409

[75] Huen I Morris DM Wright C Parker GJ Sibley CP Johnstone ED. R1 and r2* changes in the human placenta in response to maternal oxygen challenge. Magn Reson Med (2013) 70(5):1427–33. doi: 10.1002/mrm.24581 23280967

[76] Zaharchuk G Busse RF Rosenthal G Manley GT Glenn OA Dillon WP. Noninvasive oxygen partial pressure measurement of human body fluids in vivo using magnetic resonance imaging. Acad Radiol (2006) 13(8):1016–24. doi: 10.1016/j.acra.2006.04.016 16843855

[77] Muir ER Zhang Y San Emeterio Nateras O Peng Q Duong TQ. Human vitreous: Mr imaging of oxygen partial pressure. Radiology (2013) 266(3):905–11. doi: 10.1148/radiol.12120777 PMC3579170 23220896

[78] von Knobelsdorff-Brenkenhoff F Prothmann M Dieringer MA Wassmuth R Greiser A Schwenke C. Myocardial t1 and t2 mapping at 3t: Reference values, influencing factors and implications. J Cardiovasc Magn Res (2013) 15(1):1–11. doi: 10.1186/1532-429X-15-53 PMC3702448 23777327

[79] Ramadan S Paul N Naguib HE. Standardized static and dynamic evaluation of myocardial tissue properties. BioMed Mater (2017) 12(2):025013. doi: 10.1088/1748-605x/aa57a5 28065929

[80] McKee CT Last JA Russell P Murphy CJ. Indentation versus tensile measurements of young’s modulus for soft biological tissues. Tissue Eng Part B Rev (2011) 17(3):155–64. doi: 10.1089/ten.teb.2010.0520 PMC3099446 21303220

[81] Miller CC. The stokes-einstein law for diffusion in solution. Proc R Soc London Ser A Containing Pa Math Phys Character (1924) 106(740):724–49. doi: 10.1109/TBME.2017.2764111

[82] Berg HC. Random walks in biology. Princeton, New Jersey: Princeton University Press (2018).

[83] Balinov B Söderman O. Emulsions-the nmr perspective. In: Encyclopedic handbook of emulsion technology New York: Marcel Dekker (2001). p. 279–303.

