# Erratum: Perfluorocarbon emulsion enhances MR-ARFI displacement and temperature *in vitro*: Evaluating the response with MRI, NMR, and hydrophone

**DOI:** 10.3389/fonc.2023.1170338

**Published:** 2023-03-02

**Authors:** Frontiers Production Office

**Affiliations:** Frontiers Media SA, Lausanne, Switzerland

**Keywords:** HIFU, hydrophone, emulsion, sonosensitizers, MR-ARFI, colloids, perfluorocarbons (PFCs), cavitation

An omission to the funding section of the original article was made in error. The following sentence has been added: “Open access funding was provided by the University of Geneva”.

The original version of this article has been updated.

