# Supplementary material for: Perfluorocarbon emulsion enhances MR-ARFI displacement and temperature in vitro: Evaluating the response with MRI, NMR, and hydrophone
Source: Front Oncol. 2023 Jan 13;12:1025481. doi: 10.3389/fonc.2022.1025481 (PMC9880467; doi:10.3389/fonc.2022.1025481)
Supplement: Supplementary file 1 [file DataSheet_1.zip › Supplemental Materials/ARFI_gel_preparation_01072021.pdf]

Lecithin-PFOB & FTAC-PFOB Gel Preparation

01.07.2021 Gel Preparation

Gel	Surfactant	Gel %v:w of 2% v:v Emulsion	Volume of emulsion to add, mL	Volume droplets in emulsion volume, mL	86 %w:w DI H2O, mass g	Stock Solution Water convent	Water added with emulsions, mL	3 %w:w Gelatine, mass g	6 %w:w Starch, mass g	5 %w:w Powdered Milk, mass g	Total Weight, mass g	Concentrated Control gel to add, g	Sodium Azide, g
1	N/A	0,00	0,00	0,00	121,13	94,41	26,72	4,5	9,00	7,50	142,50	115,41	0.03
2	N/A	0,00	0,00	0,00	121,13	94,41	26,72	4,5	9,00	7,50	142,50	115,41	0.03
3	N/A	0,00	0,00	0,00	121,13	94,41	26,72	4,5	9,00	7,50	142,50	115,41	0.03
4	1.61% Lecithin	0,05	4,43	0,07	116,68	94,41	22,27	4,5	9,00	7,50	142,57	115,41	0.03
5	1.61% Lecithin	0,10	8,85	0,14	112,22	94,41	17,81	4,5	9,00	7,50	142,65	115,41	0.03
6	1.61% Lecithin	0,20	17,70	0,29	103,32	94,41	8,91	4,5	9,00	7,50	142,82	115,41	0.03
7	1.61% Lecithin	0,30	26,55	0,43	94,41	94,41	0,00	4,5	9,00	7,50	143,00	115,41	0.03
1	N/A	0,00	0,00	0,00	121,13	94,41	26,72	4,5	9,00	7,50	142,50	115,41	0.03
2	N/A	0,00	0,00	0,00	121,13	94,41	26,72	4,5	9,00	7,50	142,50	115,41	0.03
3	N/A	0,00	0,00	0,00	121,13	94,41	26,72	4,5	9,00	7,50	142,50	115,41	0.03
8	1.65% F_8TAC_{18}	0,05	4,32	0,07	117,57	94,41	23,16	4,5	9,00	7,50	142,57	115,41	0.03
9	1.65% F_8TAC_{18}	0,10	8,64	0,14	114,01	94,41	19,60	4,5	9,00	7,50	142,65	115,41	0.03
10	1.65% F_8TAC_{18}	0,20	17,27	0,29	106,88	94,41	12,47	4,5	9,00	7,50	142,82	115,41	0.03
11	1.65% F_8TAC_{18}	0,30	25,91	0,43	99,76	94,41	5,35	4,5	9,00	7,50	143,00	115,41	0.03
Total:			113,7	1,85	1591,6	1321,7	269,9	63	126	105	1997,1	1615,7	0.60

1. Degass 2.0 Litres of Deionized H_2O at 40 mbar for 30 minutes. During this time, label all containers to identify the gels. Use plastic cups of about 200 to 400 mL. Also, this time we need to use a plastic film at the bottom of the cups. Use two cups for each gel.
2. After degassing, add 1321,74 grams of degassed and deionized water to glass container that is compatible with stirrer and Curie bath.
3. Add 105 grams of powdered milk to large flask for use with Curie bath.
4. add 126 grams of starch to large flask for use with Curie bath.
5. Add 1000 grams/mL of degassed and deionized water to large flask for curie bath. Stir to dissolve the milk and starch.
6. add 63 grams of gelatine to 400 mL beaker. Fill the glass beaker with 321.7 grams/mL of the degassed and deionized water let gelatine soak for about 10 minutes. After soaking for 10 minutes, add gelatine and the water used to soak the gelatine to the flask for use with the Curie bath.
7. Heat while covered, with stirring, to about 90 °C. Prevent components from excessive heating by keeping the beaker from being in direct contact with the metal surface inside the oil bath. Dissolving the starch in the flask with manual stirring before placing in Curie bath has given the best results. Also, perhaps adjust the stirring rod speed.
8. Add 600 mg of sodium azide to solution while stirring.
9. Add about 60 mL of water to a separate cup and add one full pipette of methylene blue until the solution is quite dark. Makes about 2.5 mL or 2.5 g of Dye + H2O per cup.
10. For the lecithin samples, add 4.3 mL, 8.85 mL, 17.70 mL, and 26.55 mL of 1.61% Lecithin solution to the 0.05 %, 0.10%, 0.20%, 0.3% containers, respectively. Add 22.27 mL, 17.81 mL, 8.91 mL, and 0.0 mL of DG&DI H2O solution to the 0.05 %, 0.10%, 0.20%, 0.3% containers, respectively.
11. Then, add 115.32 mL of stock solution to 4 separate 400 mL beakers, and let cool to about 50-60 °C at room temperature. Add each of the lecithin-PFOB samples from the cups to separate beakers. Manually stir solution until dye is homogeneous and do not let air bubbles form. Let solution cool at room temperature for about 30-60 minutes, allowing residual air bubbles move to surface. Then, cover, put in ice bath to solidify, then store in the fridge.
12. For the FTAC gels, add 4.32 mL, 8.64 mL, 17.27 mL, and 25.69 mL of 1.65% F_8TAC_{18} solution to the 0.05%, 0.10%, 0.20%, 0.3% containers, respectively. Add 23.16 mL, 19.60 mL, 12.47 mL, and 5.35 mL of DG&DI H2O solution to the 0.05 %, 0.10%, 0.20%, 0.3% containers, respectively.
13. Then, add 115.32 mL of stock solution to 4 separate 400 mL beakers, and let cool to about 50-60 °C at room temperature. Add each of the lecithin-PFOB samples from the cups to separate beakers. Manually stir solution until dye is homogeneous and do not let air bubbles form. Let solution cool at room temperature for about 30-60 minutes, allowing residual air bubbles move to surface. Then, cover, put in ice bath to solidify, then store in the fridge.
14. For control gel, add 115.32 mL of stock solution, add 26.72 mL of DG&DI H2O, and add one pipette of the dye solution. Manually stir solution until dye is homogeneous and do not let air bubbles form. Cover and put in fridge.

Mixing beaker mass, g:

Mixing beaker mass with Reagents, before heating, mass, g:

Mixing beaker mass with Reagents, after heating, mass, g:

Solution weight after mixing:

*Volume off emulsion to add calculated with following equation:

$$Vadd = \frac{Cdes * Vi}{Cem}$$

Vadd: volume of emulsion stock solution to add to stock solution, Cdes: Desired final concentration (v:v) of emulsions in gels, Cem: stock solution concentration of emulsions (~2%), Vi: Initial volume of liquid gel. This is taken to be the mass of the gel solution since it is composed primarily of water.

*Note. We generally want to maintain two controls gels for each experiment. Plan for 3 to allow room for error in the preparation. For instance if the final amount of stock solution is less than expected due to water evaporation during heating.

*Note: Gels need to be made more concentrated initially. Then, the water in the emulsion solution will be used to dilute the gels to their proper concentration.
