# Supplementary figures and images for: Perfluorocarbon emulsion enhances MR-ARFI displacement and temperature in vitro: Evaluating the response with MRI, NMR, and hydrophone
Source: Front Oncol. 2023 Jan 13;12:1025481. doi: 10.3389/fonc.2022.1025481 (PMC9880467; doi:10.3389/fonc.2022.1025481)

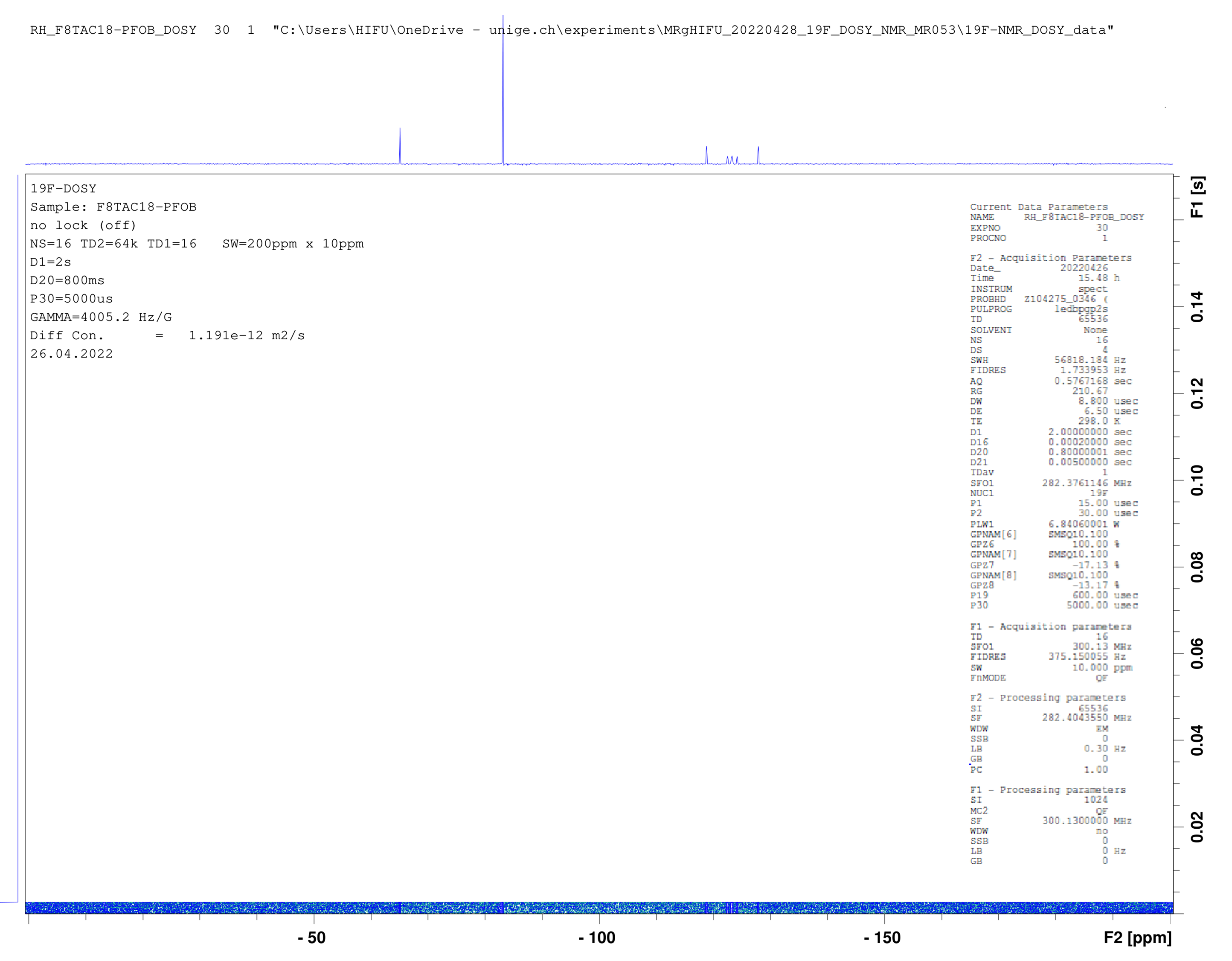

Supplement: Supplementary file 1 [file DataSheet_1.zip › Supplemental Materials/19f_nmr_dosy_ftac_pfob.png]

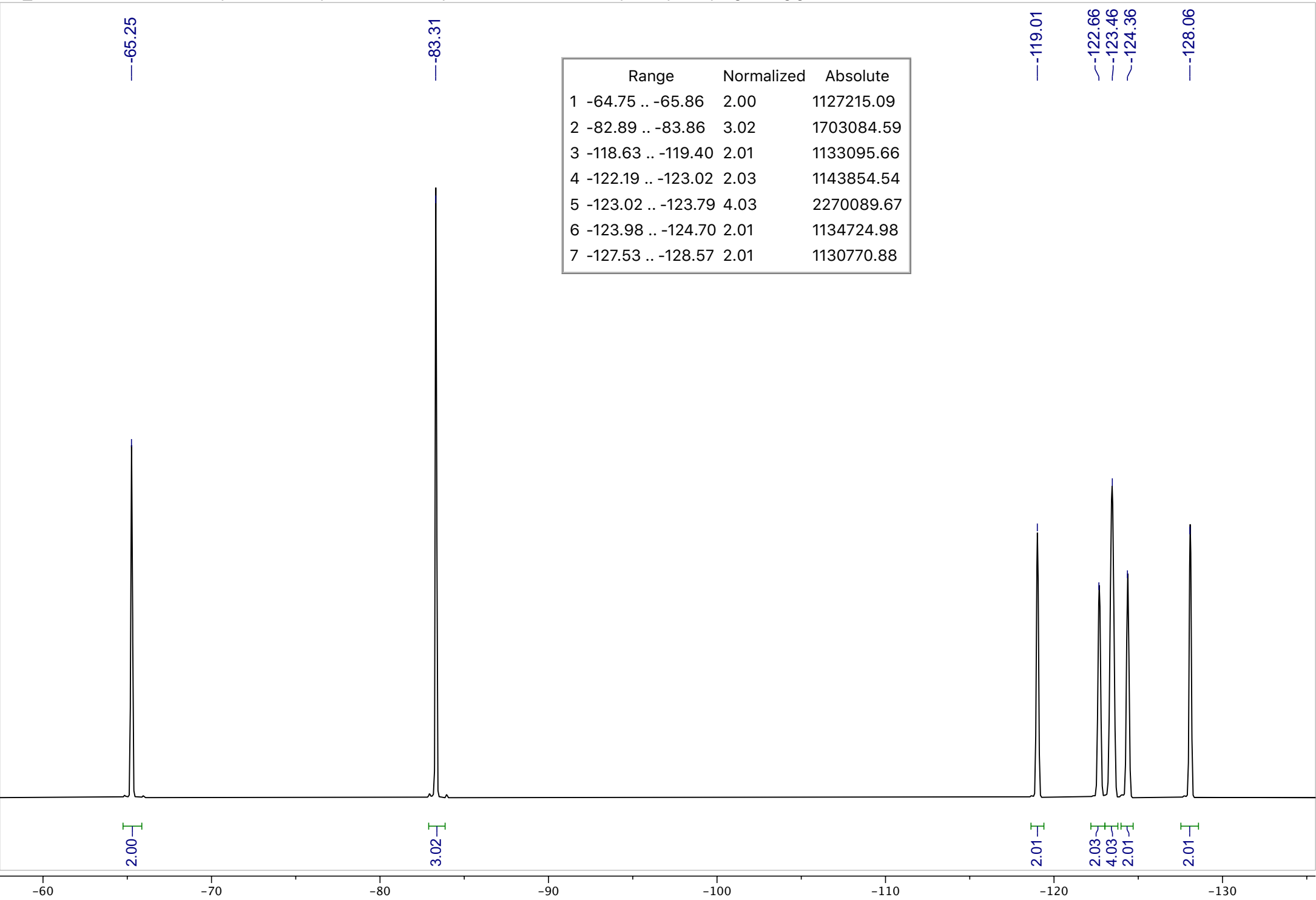

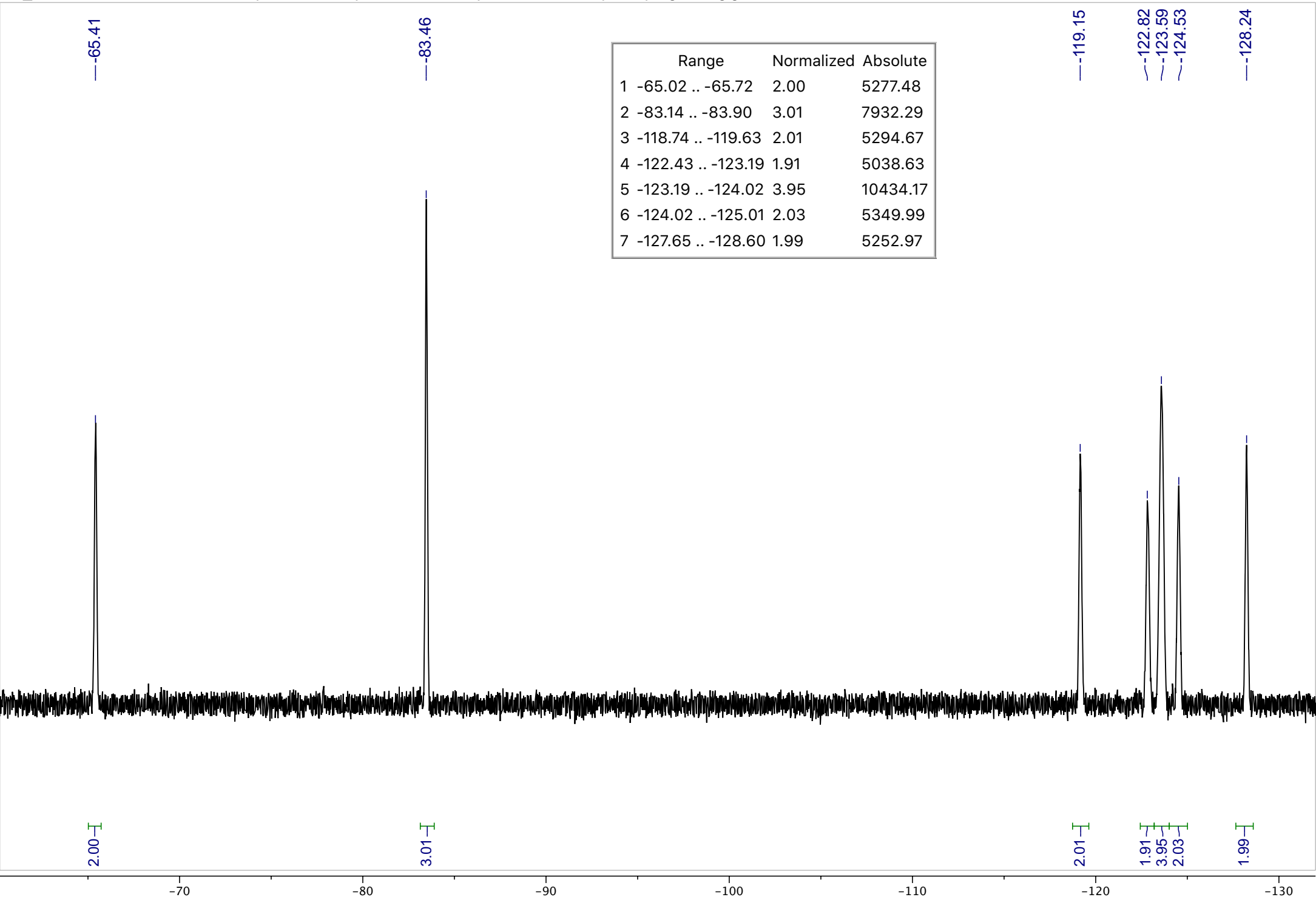

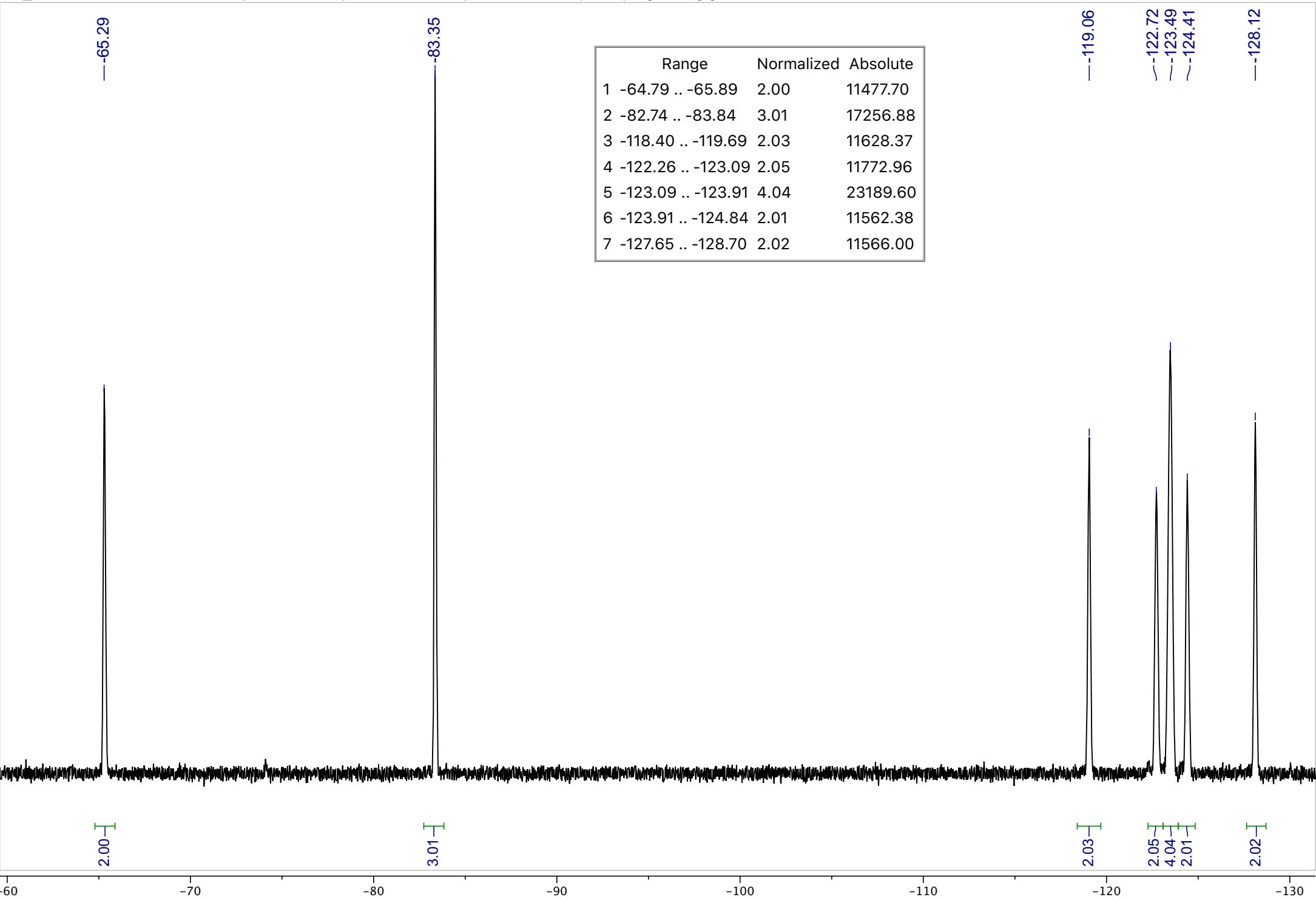

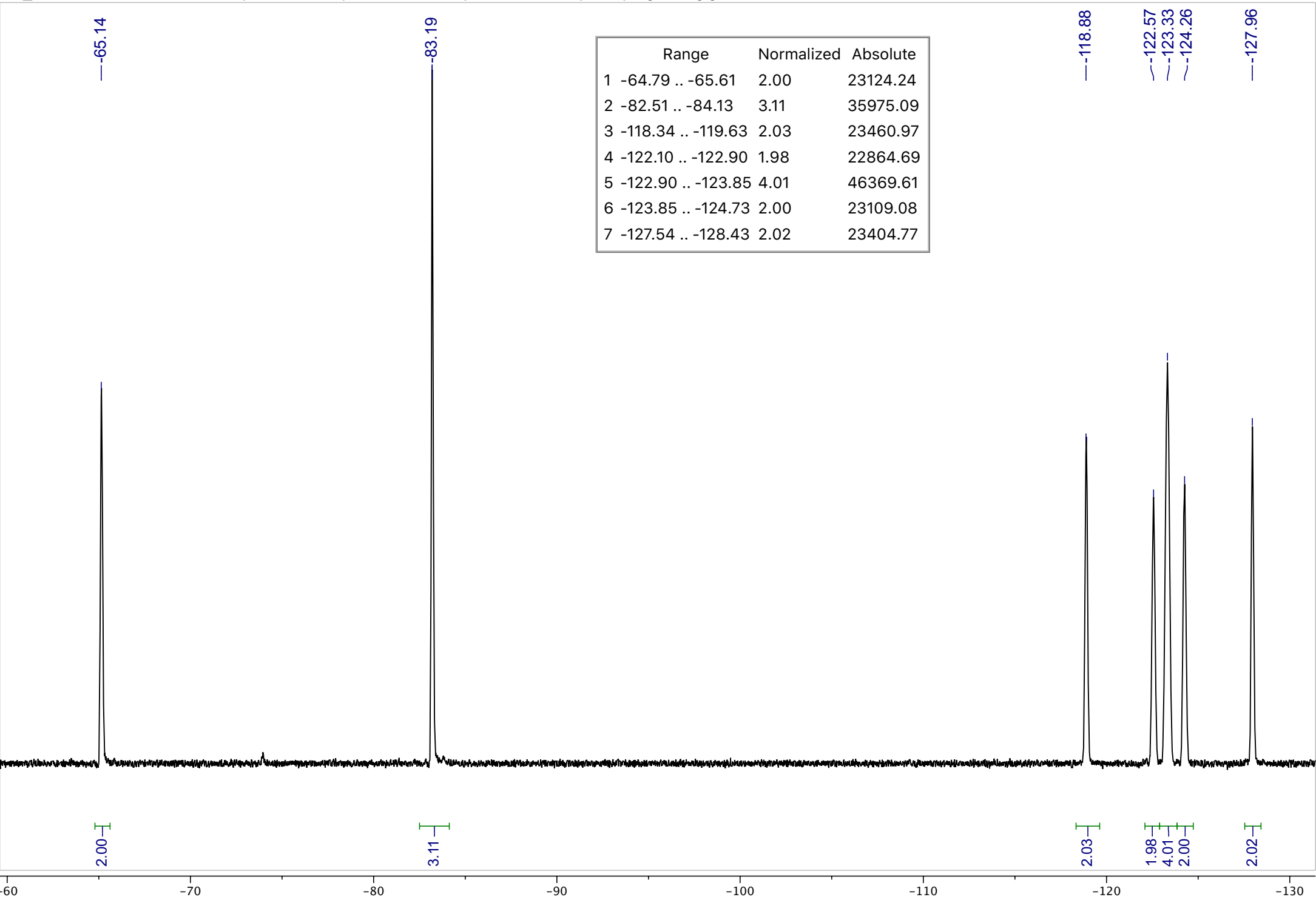

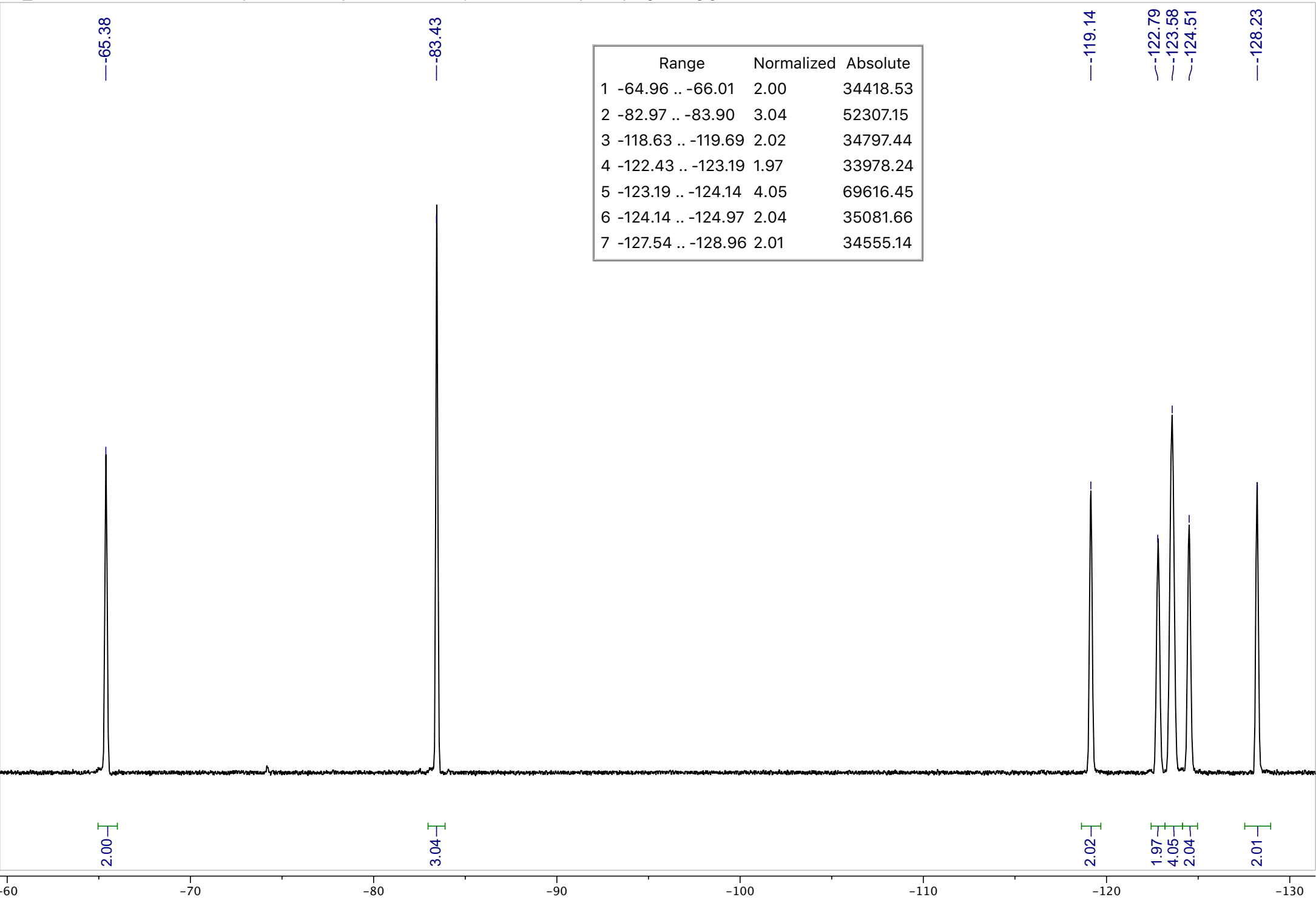

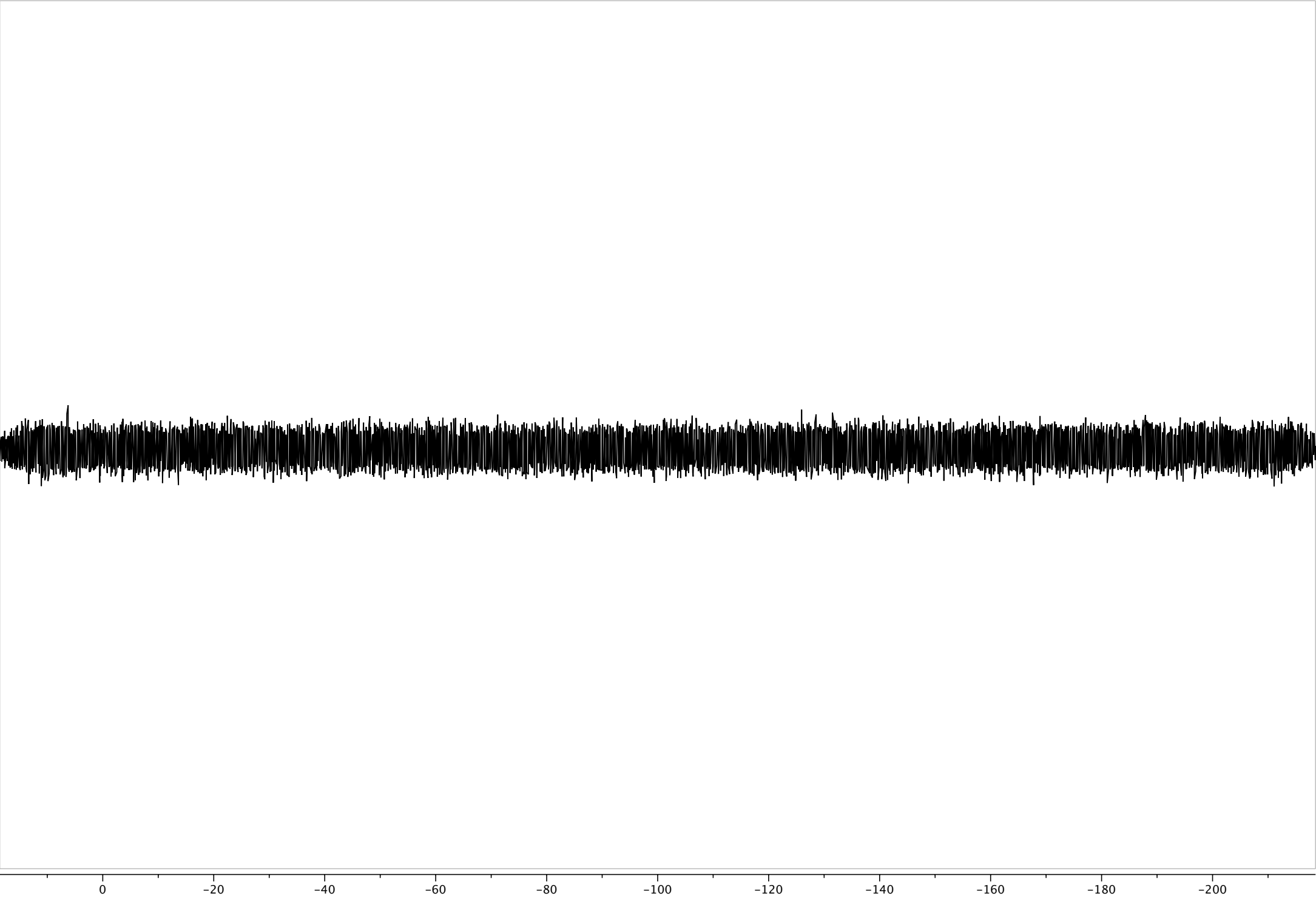

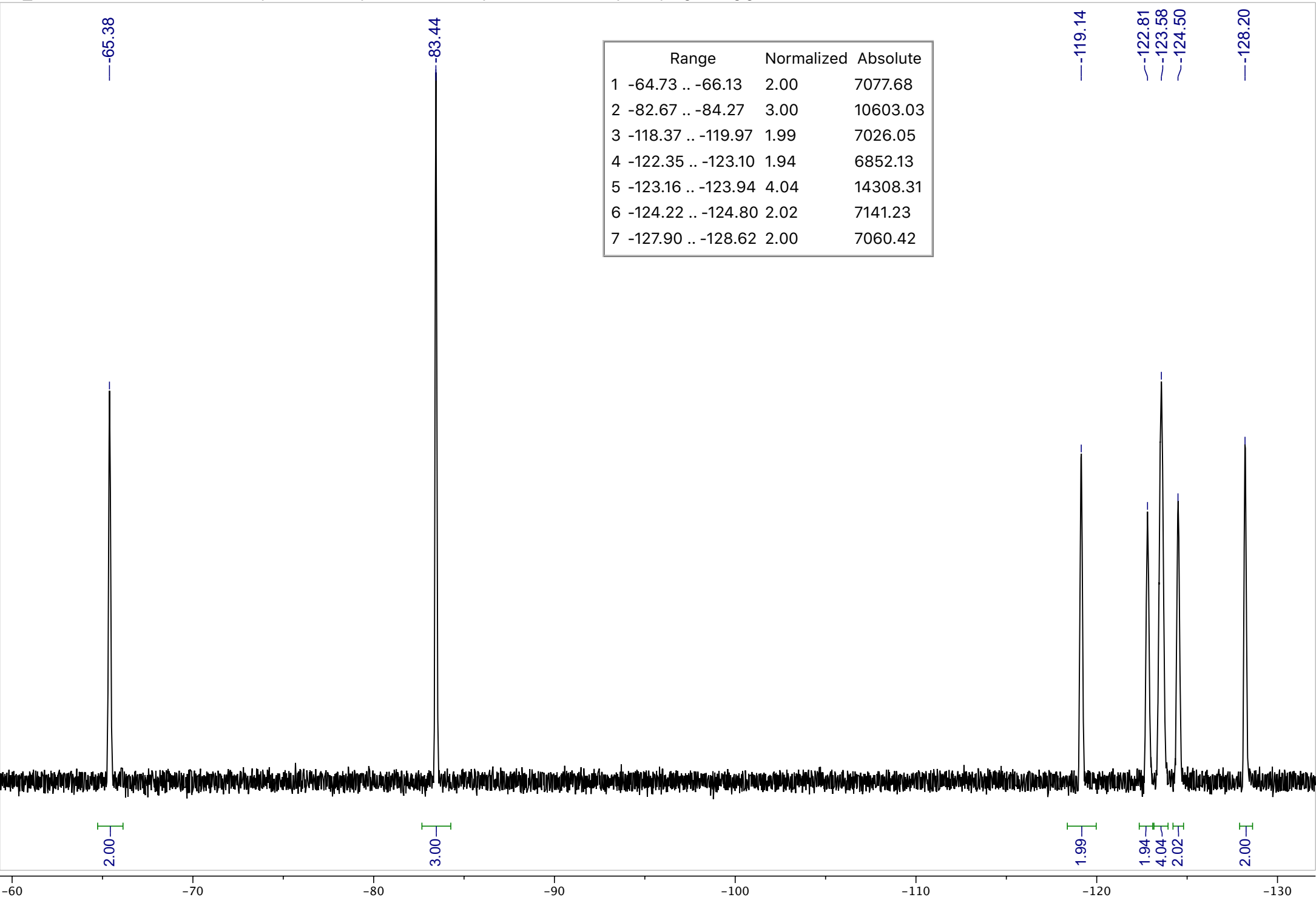

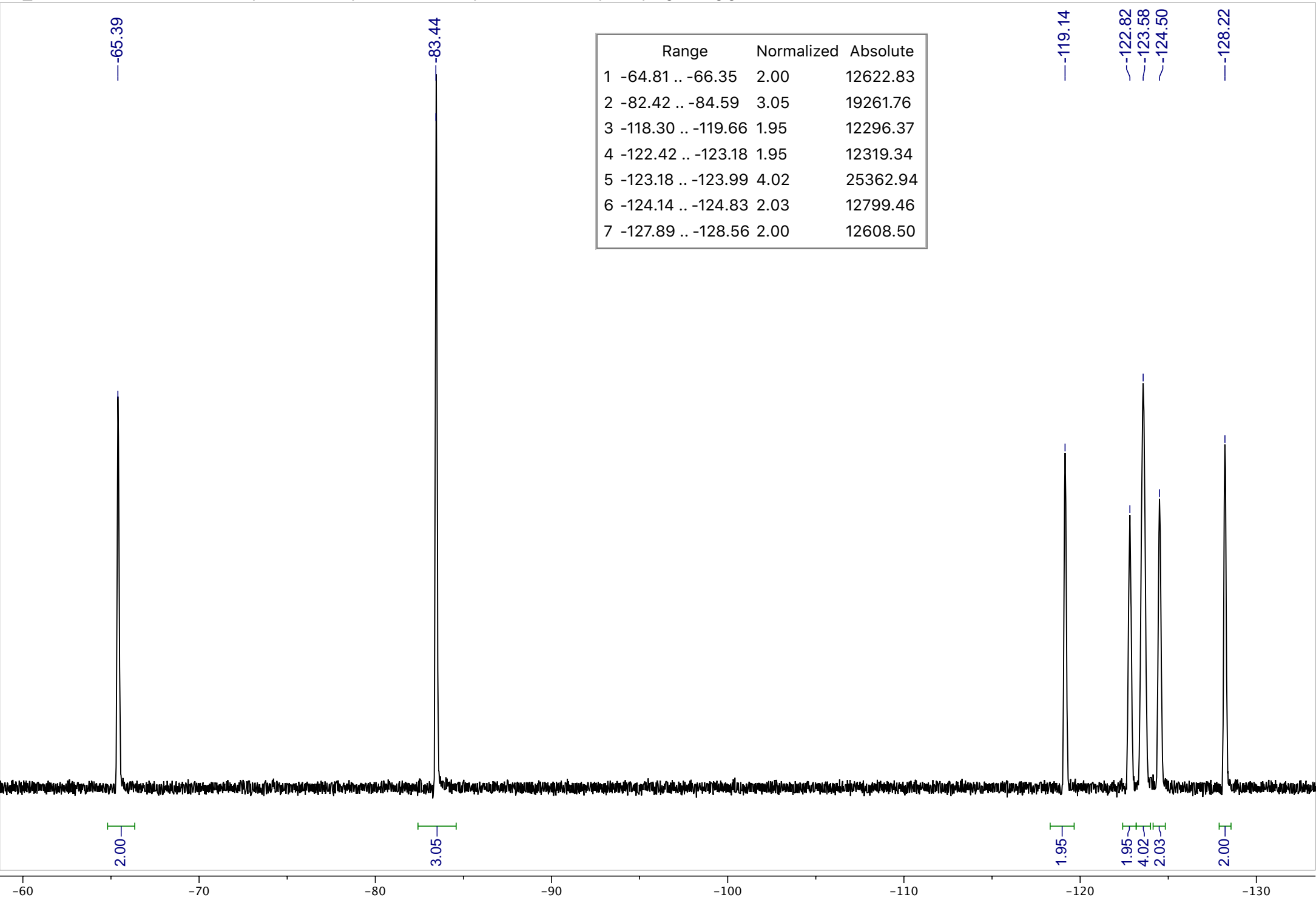

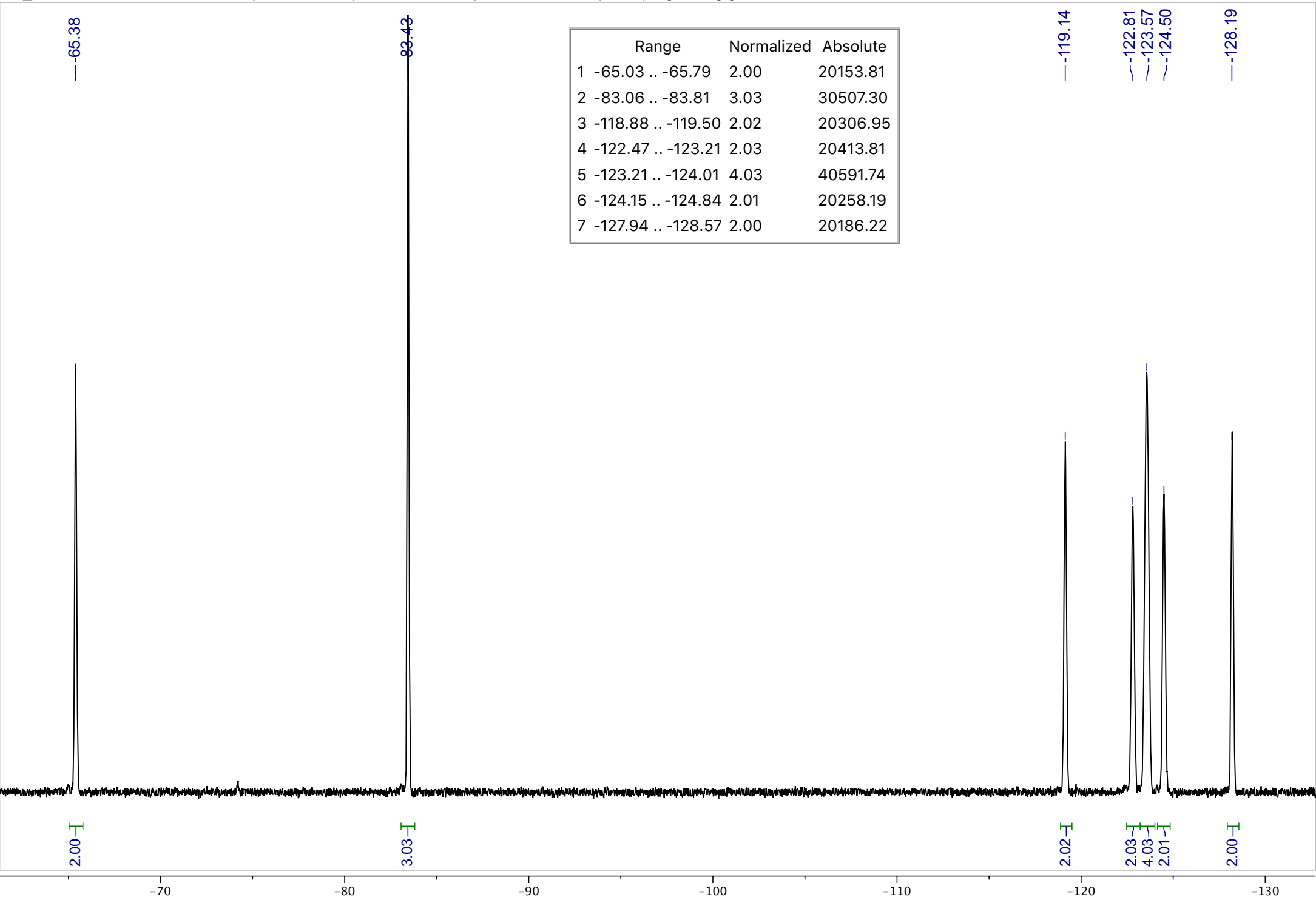

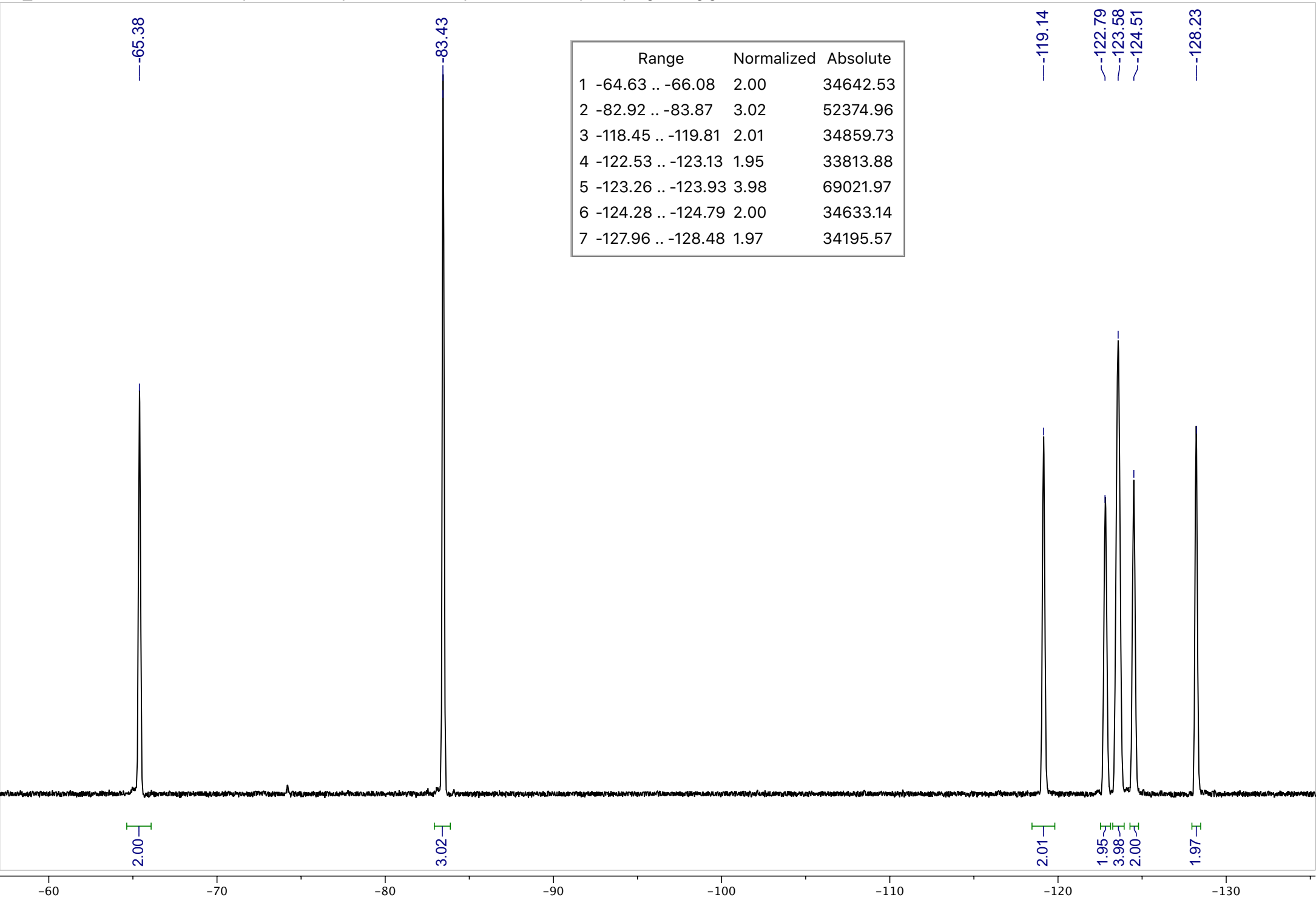

Supplement: Supplementary file 1 [file DataSheet_1.zip › Supplemental Materials/perflubron_19F_NMR.pdf]
